# Mind the leaf anatomy while taking ground truth with portable chlorophyll meters

**DOI:** 10.1038/s41598-024-84052-5

**Published:** 2025-01-13

**Authors:** Zuzana Lhotáková, Eva Neuwirthová, Markéta Potůčková, Lucie Červená, Lena Hunt, Lucie Kupková, Petr Lukeš, Petya Campbell, Jana Albrechtová

**Affiliations:** 1 Department of Plant Experimental Biology, Faculty of Science, Charles University, Viničná 5, 12800 Prague, Czech Republic; 2 Department of Applied Geoinformatics and Cartography, Faculty of Science, Charles University, Albertov 6, 12800 Prague, Czech Republic; 3 Global Change Research Institute of the Czech Academy of Sciences, Bělidla 986/4a, 60300 Brno, Czech Republic; 4 University of Maryland Baltimore County and NASA/Goddard Space Flight Center, Code 618, Greenbelt, MD 20771 USA

**Keywords:** Chlorophyll, Leaf structure, Leaf pigments, Leaf with hypodermis, Vegetation index, Remote sensing, Plant sciences, Ecophysiology, Forestry, Environmental monitoring

## Abstract

A wide range of portable chlorophyll meters are increasingly being used to measure leaf chlorophyll content as an indicator of plant performance, providing reference data for remote sensing studies. We tested the effect of leaf anatomy on the relationship between optical assessments of chlorophyll (Chl) against biochemically determined Chl content as a reference. Optical Chl assessments included measurements taken by four chlorophyll meters: three transmittance-based (SPAD-502, Dualex-4 Scientific, and MultispeQ 2.0), one fluorescence-based (CCM-300), and vegetation indices calculated from the 400–2500 nm leaf reflectance acquired using an ASD FieldSpec and a contact plant probe. Three leaf types with different anatomy were included: dorsiventral laminar leaves, grass leaves, and needles. On laminar leaves, all instruments performed well for chlorophyll content estimation (R^2^ > 0.80, nRMSE < 15%), regardless of the variation in their specific internal structure (mesomorphic, scleromorphic, or scleromorphic with hypodermis), similarly to the performance of four reflectance indices (R^2^ > 0.90, nRMSE < 16%). For grasses, the model to predict chlorophyll content across multiple species had low performance with CCM-300 (R^2^ = 0.45, nRMSE = 11%) and failed for SPAD. For Norway spruce needles, the relation of CCM-300 values to chlorophyll content was also weak (R^2^ = 0.45, nRMSE = 11%). To improve the accuracy of data used for remote sensing algorithm development, we recommend calibration of chlorophyll meter measurements with biochemical assessments, especially for species with anatomy other than laminar dicot leaves. The take-home message is that portable chlorophyll meters perform well for laminar leaves and grasses with wider leaves, however, their accuracy is limited for conifer needles and narrow grass leaves. Species-specific calibrations are necessary to account for anatomical variations, and adjustments in sampling protocols may be required to improve measurement reliability.

## Introduction

Chlorophyll content is arguably the most important photosynthetic indicator of vegetative function and physiological condition^1^. Chlorophyll (Chl) absorbs photosynthetically active radiation indicating vegetation´s photosynthetic potential, ecosystem function, and productivity^2,3^. Chl serves as a non-specific vegetation stress marker for monitoring vegetation’s response to climate change, anthropogenic pollution, or management practices^4–8^.

Remote sensing provides a low-cost, non-destructive alternative to plant in situ sampling for chlorophyll content estimation (reviewed by Kandpal and Kumar^9^). Satellite-based spatially-explicit knowledge of vegetation Chl is fundamental for managing agricultural and forest ecosystems— in situ ground surveys cannot capture the spatial heterogeneity associated with cover type and function and the temporal dynamics of these systems. Despite the advantages of remote sensing, field ground truth Chl measurement is necessary for its retrieval validation^10,11^ and development of suitable Chl retrieval workflows from spectral data^12^.

Chl content determines leaf optical properties—absorption, transmittance, and reflectance—in the visible (VIS) part of the electromagnetic spectrum^13^. Chlorophyll a and b pigments have unique absorption spectra with strong peaks in the blue (428–453 nm) and red (640–660 nm) spectral regions according to the solvent used for extraction^14^, which shift to longer wavelengths (up to 500 nm in blue and 680 nm in red) due to association with proteins of chloroplast membranes and cellular structure of leaf tissues^15,16^. Thus, the leaf reflectance and transmittance are very low in blue and red, but sharply increase in the near-infrared (NIR) spectral range, where the radiation is not absorbed by Chl molecules but rather scattered on leaf surface and cell wall to air interface^13^ within the leaf. The radiation incident on a leaf is also scattered and reflected on its surface structures, such as waxes and trichomes^17–19^. After the photon absorption by chlorophyll molecules, part of the energy is emitted as fluorescence: weak radiation in the red and far-red wavelengths^15^. The red fluorescence (around 680 nm) is strongly reabsorbed within the mesophyll, particularly within the leaves with higher chlorophyll content per leaf area. By contrast, far-red fluorescence (approx. 700–740 nm, beyond the Chl absorption maxima mentioned above) is much less reabsorbed and thus less sensitive to leaf chlorophyll content^20^.

Anthocyanins, protective pigments, also influence leaf optical properties in VIS^21,22^, particularly during autumnal senescence and Chl degradation^23^. Despite their primary absorption maxima in UV (280–320 nm) and green (490–550 nm)^24^, anthocyanins exhibit absorption even in red wavelengths (600–630 nm)^25,26^. At high leaf concentrations, anthocyanins contribute to light absorption even in the longer wavelengths (600–650 nm)^27^. The interference of Chl and anthocyanin absorption in the green spectral region may influence anthocyanin detection using absorbance- and reflectance-based techniques ^16^.

Various portable devices have been developed for the non-destructive optical assessment of Chl content^9^. Most portable chlorophyll meters measure the proportion of radiation not absorbed by chlorophyll at specific absorption wavelengths, which is inversely related to leaf chlorophyll content. The leaf transmittance or reflectance are typically recorded at two different wavelengths: the “index band”, where transmittance (and reflectance) are more sensitive to changes in leaf Chl content, and the “reference band”, where transmittance (and reflectance) remain stable regardless of changes in Chl content^28–30^. Transmittance-based instruments (e.g., Konica Minolta SPAD-502 + , Opti-Sciences CCM-200, PhotosynQ MultispeQ and atLEAF CHL PLUS) typically provide accurate pigment content estimates (82–95%) for plant species with laminar, dorsiventral leaves^31^. There are also instruments designed to measure leaf Chl content and other leaf pigments, such as flavonols and anthocyanins, by analysing their screening effect on Chl fluorescence^32,33^. Chl fluorescence can also be used for optical Chl estimation, as far-red to red Chl fluorescence ratio is positively correlated with leaf Chl content^15,34,35^. The general limitations of band-based analyses and vegetation indices such as case-specificity and the most sensitive wavelength selection^36^ also apply to portable chlorophyll meters.

Transmittance-based methods rely on light passing through the leaf, while reflectance-based methods measure the light reflected from the leaf surface and internal tissues. Therefore, both optical variables can be influenced by the leaf’s internal structure. Fluorescence-based techniques detect chlorophyll fluorescence emission, which can vary with leaf thickness and structure. Transmittance- and reflectance-based Chl measurement techniques require the field of view (FOV) of the instrument to be fully covered by the leaf, which can be challenging for conifer needles or narrow grass leaves. Fluorescence-based techniques, such as those used by the CCM-300 (OptiSciences, Inc., Hudson, NH, USA), measure Chl fluorescence, using an optical fibre with a very small field of view (7 mm^2^). However, FOV constraints still exist in fluorescence-based methods due to different phenomena, such as the uneven distribution of fluorescence signals.

The leaf biophysical (leaf mass per area–LMA, equivalent water thickness–EWT)^37^ and anatomical traits (density of mesophyll, leaf thickness^38^, cuticle thickness^39^, epidermal cell shape, and surface characteristics^40^) and the distribution of chloroplasts affect the path of light in the leaf and thus, determine its optical properties^13,41^. The adaxial-abaxial polarity of the leaf is regulated by a gene network^42,43^, partly species-specific^44,45^ and influenced by many environmental factors (e.g., radiation intensity^46^, water availability^47^). Leaf thickness may affect non-destructive Chl detection^48^, as light is more scattered in thicker leaves^49^. Uneven chlorophyll distribution and light scattering by non-absorbing cell components alter the light path through the leaf. These effects have been described as the sieve effect and the detour effect, respectively^50^. The sieve effect refers to the lower absorption of photosynthetic wavelengths by the leaf compared to a homogeneous solution of proteins and pigments. It is caused by the intracellular localization of pigments within chloroplasts as well as light-dependent movements of chloroplasts^51^. The detour effect refers to an increase in the light path length for the weakly absorbed parts of the spectrum^50,52^. The detour effect is caused by light scattering at the cell wall-to-air space interface, leading to increased light absorption and reflection, and decreased transmittance, particularly in the NIR^30,50,52^. Light scattering within the leaf affects the relationship between absolute and optically assessed chlorophyll content, especially in leaves with very low and very high chlorophyll content^35,53^. When interpreting fluorescence-based measurements, reabsorption of red fluorescence must be considered, as it can lead to underestimation of chlorophyll content in thicker leaves^35^.

**Fig. 1:**
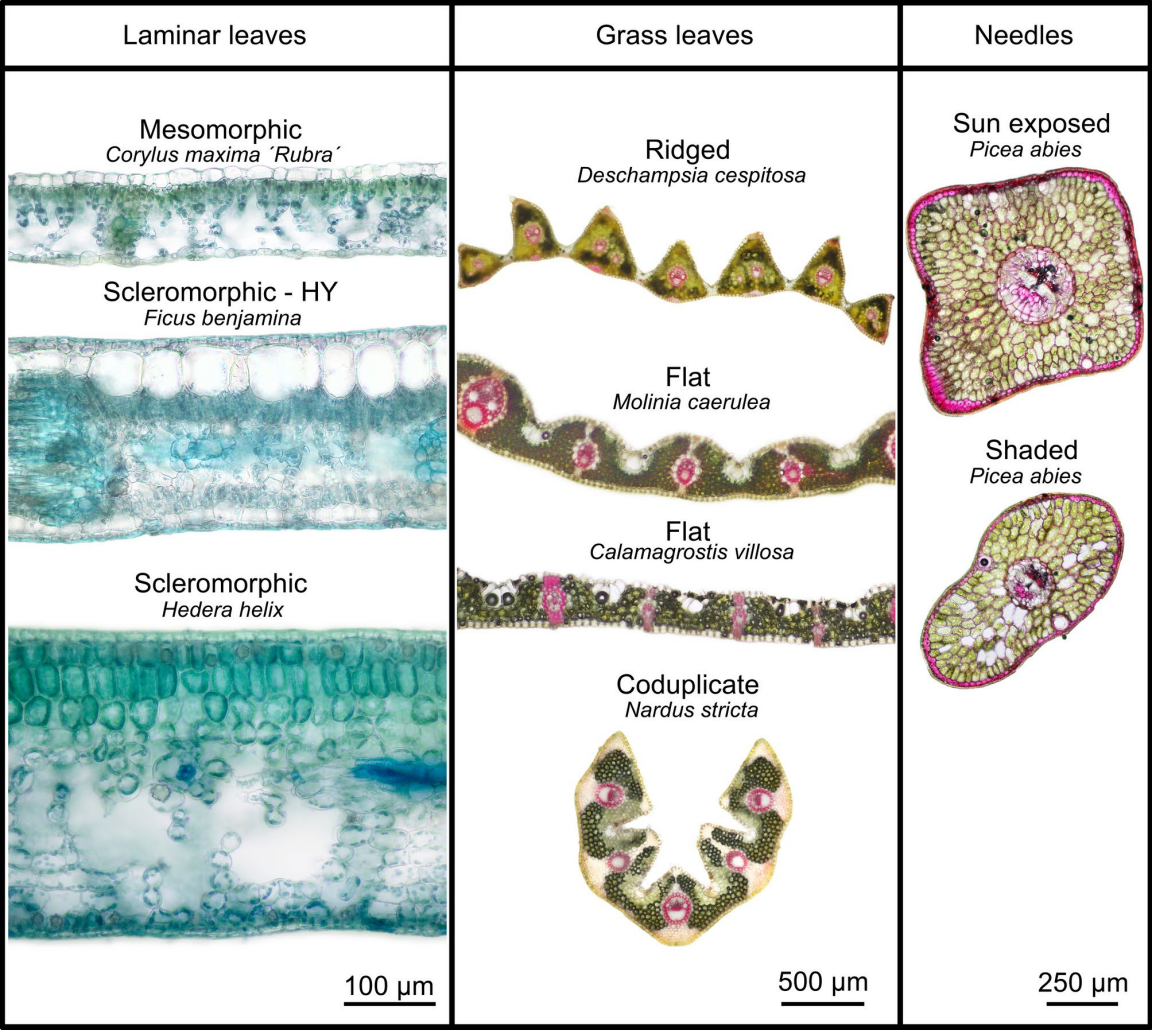
Examples of leaf internal structure on cross sections of three anatomically distinctive leaf types: Laminar, dorsiventral leaves—left column (mesomorphic—leaves of deciduous tree species represented by *Corylus maxima* ‘Rubra’; scleromorphic leaves with pronounced hypodermis (HY) represented by *Ficus benjamina*, and scleromorphic leaves represented by *Hedera helix*, note the thick cuticle on adaxial epidermis and well-developed palisade parenchyma (stained with Toluidine blue)). Grass leaves—middle column, all four species under study, stained with phloroglucinol (cherry red reaction with lignin). Needles—right column represented by *Picea abies*, sun-exposed and shaded needles; stained by phloroglucinol (cherry red reaction with lignin). Hand-microtome sections, bright field, light microscopy. Scale bars dimensions indicated. Original photos of studied specimens taken by authors.

Numerous studies have examined the factors influencing the relationship between biochemically and optically assessed leaf Chl content in a wide range of plant species, typically with laminar, dorsiventral leaves (e.g.,^16,28,30–32,45,53–55^). Less attention has been given to the effect of leaf structure on the relationship between biochemical and optical Chl assessment. This study aims to explore how leaf anatomy and biophysical traits influence the Chl measurement reliability of four tested chlorophyll meters: SPAD-502, Dualex-4 Scientific, and MultispeQ 2.0, and CCM-300. We focused on three types of leaves with differing anatomy: (1) laminar dorsiventral leaves of dicotyledonous species, (2) grass leaves of monocotyledonous species, and (3) conifer needles (Fig. 1). To cover a wider range of laminar leaf functional adaptations with differing internal leaf structures, we included not only leaves with the typical mesomorphic anatomy of temperate deciduous trees but also leaves with scleromorphic anatomy, which have adaptations to prevent water loss, such as a dense mesophyll, a pronounced cuticle and a wax layer on the epidermis. In addition, some scleromorphic leaves have a sub-epidermal layer of large, oval hypodermal cells with a pronounced central vacuole, as seen in *Ficus lyrata*^56^ or *Ficus benjamina*^57^, which affect leaf optical properties^58^. We classified these as a special category of laminar scleromorphic leaves with a hypodermis (HY).

We tested three specific hypotheses:

H1: The transmittance- and fluorescence-based measurement techniques restrict the ability to develop broad, universally applicable relationships between optically (Chl_opt_) and laboratory-determined ‘absolute’ chlorophyll content (Chl_abs_) across different leaf anatomical types (laminar leaves, grass leaves, and needles).

H2: In laminar leaves, the optically determined Chl content (Chl_opt_) measured by four different chlorophyll meters vary with biophysical and anatomical traits (represented as LMA, EWT, tissue thickness and hypodermis presence) as well as leaf colour, which is expressed in terms of chlorophyll, carotenoid, and anthocyanin contents.

H3: The empirical models for optical-absolute prediction of leaf Chl based on readings from different portable meters (Chl_opt_) and VIs are generalizable to deciduous woody species with laminar mesomorphic leaves.

## Results

### The differences in leaf anatomy restrict the ability to develop broad, universally applicable relationships between optical and absolute leaf Chl content

**Fig. 2:**
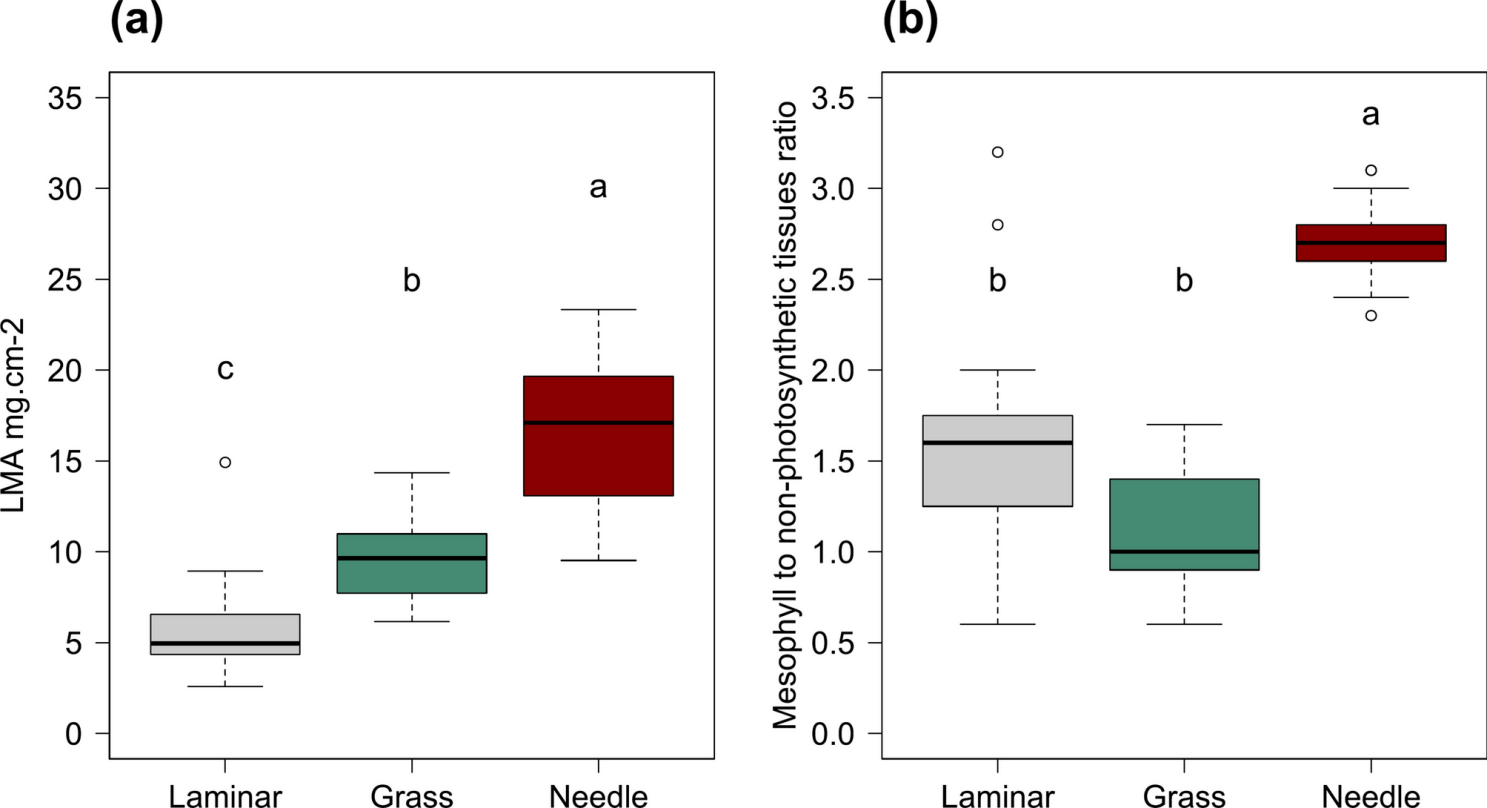
Biophysical and anatomical traits of the three studied leaf anatomical types (i.e., laminar leaves, grass leaves, and needles). Leaf mass per area (**a**). Mesophyll to non-photosynthetic tissues ratio in cross section (**b**). Different letters above boxes show significant differences at α = 0.05. Kruskal–Wallis Multiple-Comparison Z-Value Test. n = 16; 24 and 30 for laminar leaves, grass leaves, and needles, respectively.

We confirmed that the three leaf anatomical types differ in biophysical and anatomical traits such as leaf mass per area (LMA) and the ratio of mesophyll to non-photosynthetic tissues in cross-section (Fig. 2). Descriptive statistics for leaf anatomical groups regarding LMA, equivalent water thickness (EWT), and pigments are reported in Supplementary Table S1. The analysis was conducted on a subset of 16 laminar leaves, 24 grass leaves, and 30 needle samples, selected based on the availability of complete anatomical and biochemical data. Laminar leaves exhibited the lowest LMA, followed by grass leaves, with needles showing the highest LMA (Fig. 2a). In terms of mesophyll-to-non-photosynthetic tissue ratio, needles exhibited the highest ratio, followed by laminar leaves, with grasses showing the lowest (Fig. 2b).

We evaluated both linear and polynomial regression models to predict chlorophyll content based on chlorophyll meter readings. The performance of these models was compared using the coefficient of determination (R^2^), root mean square error (RMSE), and normalized RMSE (nRMSE) to quantify the accuracy and uncertainty of the predictions. The linear relationships, including R^2^ are visualized; the model equations, RMSE, and nRMSE, are primarily presented in the Supplementary Materials.

**Fig. 3:**
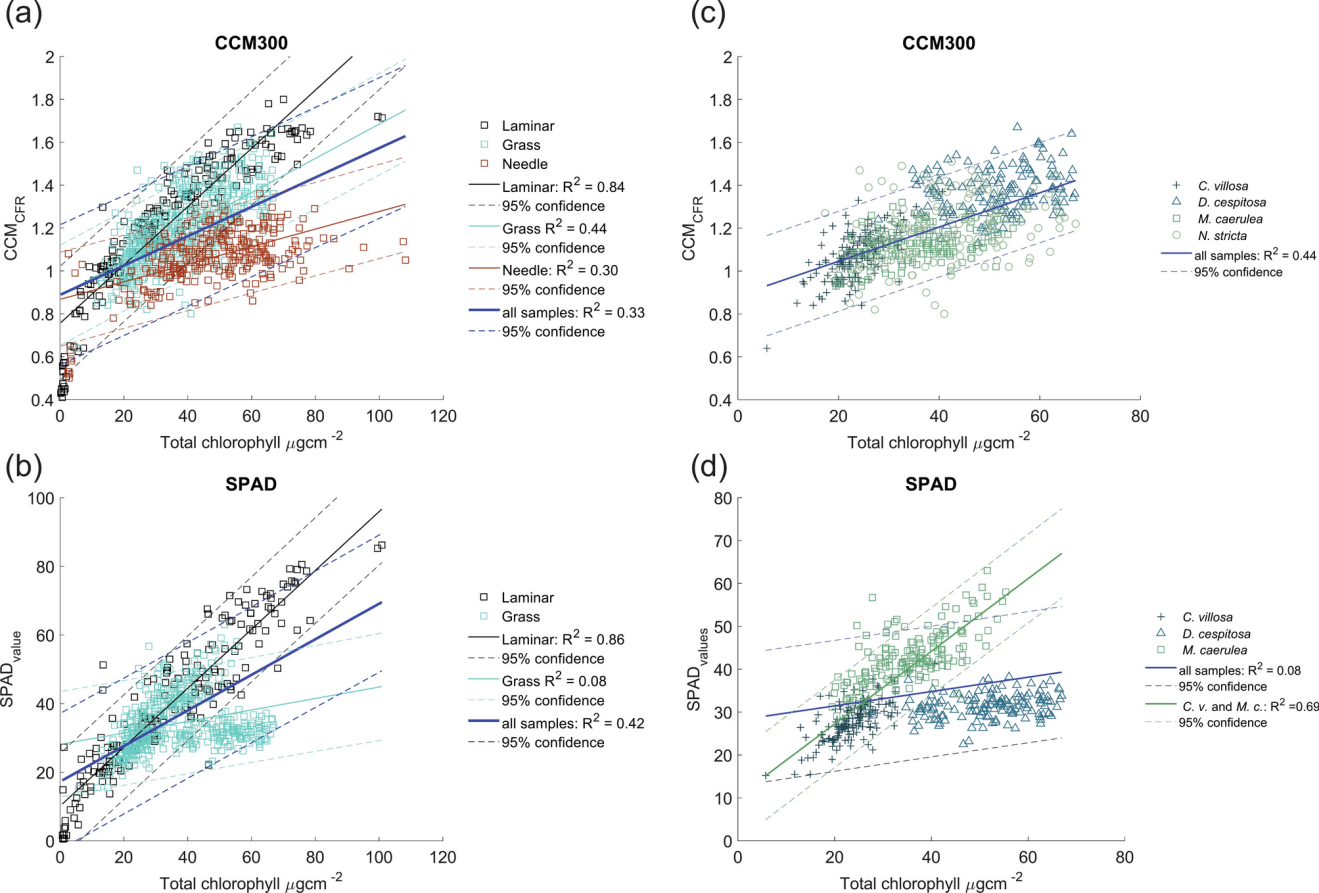
Linear relationships between Chl assessed spectrophotometrically in the lab (Total chlorophyll µg cm^−2^) and measured with chlorophyll meter CCM-300 (CCM_CFR_) for all leaf anatomical types (**a**) and grass leaves (**c**). Relationships between Chl extracted in the lab (Total chlorophyll µg.cm^-2^) and measured with chlorophyll meter SPAD (SPAD_value_) for laminar and grass leaves (**b**) and grass leaves (**d**). Thick blue line in always corresponds to the generic linear model for all included leaf types or species. Black line corresponds to linear model for laminar leaves (**a**, **b**); cyan line corresponds to linear model for grass leaves (**a**, **b**), and red line corresponds linear fit in needles (**a**). Green line in (**d**) corresponds to model for grasses wit flat leaves (*Calamagrostis villosa*—C. v. and *Molinia careulea* M. c.). Dashed lines of respective colours correspond to 95% confidence intervals.

First, generic linear models of relationship between Chl_abs_ and Chl_opt_ for all leaf anatomical types were constructed for chlorophyll meter CCM-300 (Fig. 3a). The generic model was not suitable for all leaf types together (R^2^ = 0.33) as the R^2^ for laminar leaves and grass leaves separately were higher. The ideal model would accurately predict chlorophyll content across all leaf types with high precision, but this was not achieved. Similarly, the nRMSE for the generic linear model was higher (12%) than nRMSE for laminar and grass leaves separately (9.6% and 11.5%, respectively). The polynomial models showed slightly better performance than linear ones (Supplementary Tables S2 and S3) with R^2^ = 0.92 and nRMSE 6.6% for the laminar leaf model.

Among the transmittance-based chlorophyll meters, only the SPAD meter could be used on both laminar and grass leaves (except for the narrow leaves of *Nardus stricta*, which do not fit within the small SPAD field of view) to construct a generic linear model for these two anatomical leaf types (Fig. 3b). The models performed well for laminar leaves, in contrast to grasses, where data points clustered by species (Fig. 3c, d). For spruce needles, the best model fit of Chl_opt_ and Chl_abs_ was observed for polynomial regression of CCM_values_ (R^2^ = 0.45; nRMSE = 11.3%) – the linear relationship was weaker with higher error (R^2^ = 0.30; nRMSE = 12.8%; Fig. 3a). The equations for the best fitting models and linear models shown in Fig. 3 are presented in Supplementary Tables S2 and S3.

According to our hypothesis on leaf anatomy affecting the precision and error of the chlorophyll prediction, separate models were trained for laminar leaves, grass leaves, and needles. The R^2^, RMSE, and nRMSE values of the statistical relationships between independent variables Chl_abs_ and Chl_opt_ (CCM_values_, SPAD_values_, Dx_values_, MSPQ_values_ and Chl VIs: Vogelmann, NDchl, Datt2, and RMSR) as dependent variables are shown in Supplementary Table S4. The R^2^ for linear, logarithmic, and polynomial models are shown and generally the model performance for a given Chl_opt_ and leaf type is similar for all tested models. Usually, the model performance evaluated by the nRMSE resulted in similar rankings as by R^2^. Even the relationships with low R^2^ (e.g., the *Nardus stricta* linear model; Supplementary Table S4, continuing) showed nRMSE up to 21%.

The visualizations of the model fit between Chl_abs_ and Chl_opt_ are shown for grass leaves (Fig. 3c, d) for CCM-300 and SPAD. For CCM_values_ the best performance was observed for logarithmic regression with CCM_values_ (R^2^ = 0.45; nRMSE 11.4%), the linear regression was comparable (R^2^ = 0.44; nRMSE 11.5%). For SPAD_values_ the polynomial regression performed best (R^2^ = 0.31; nRMSE 14.0%), however, the linear relationship was very weak (R^2^ = 0.08; nRMSE 16.2%). For SPAD_values_, the data strongly clustered by species, so species-specific models were constructed. The polynomial model showed the best performance for *Calamagrostis villosa* and *Molinia caeruela* (both R^2^ = 0.46; Table S5). *Calamagrostis villosa* and *Molinia caeruela* have flat leaves and very similar internal leaf structure (Fig. 1), so a common model for these two species was constructed (Fig. 3d) with R^2^ = 0.69 and nRMSE = 10.8%. However, the strong species clustering along the regression line remains evident. It was not possible to train any model for Chl_abs_ and SPAD_values_ for *Deschampsia cespitosa*. The equations for the best fitting and linear models for grasses shown in Fig. 3c, d are presented in Supplementary table S5.

**Fig. 4:**
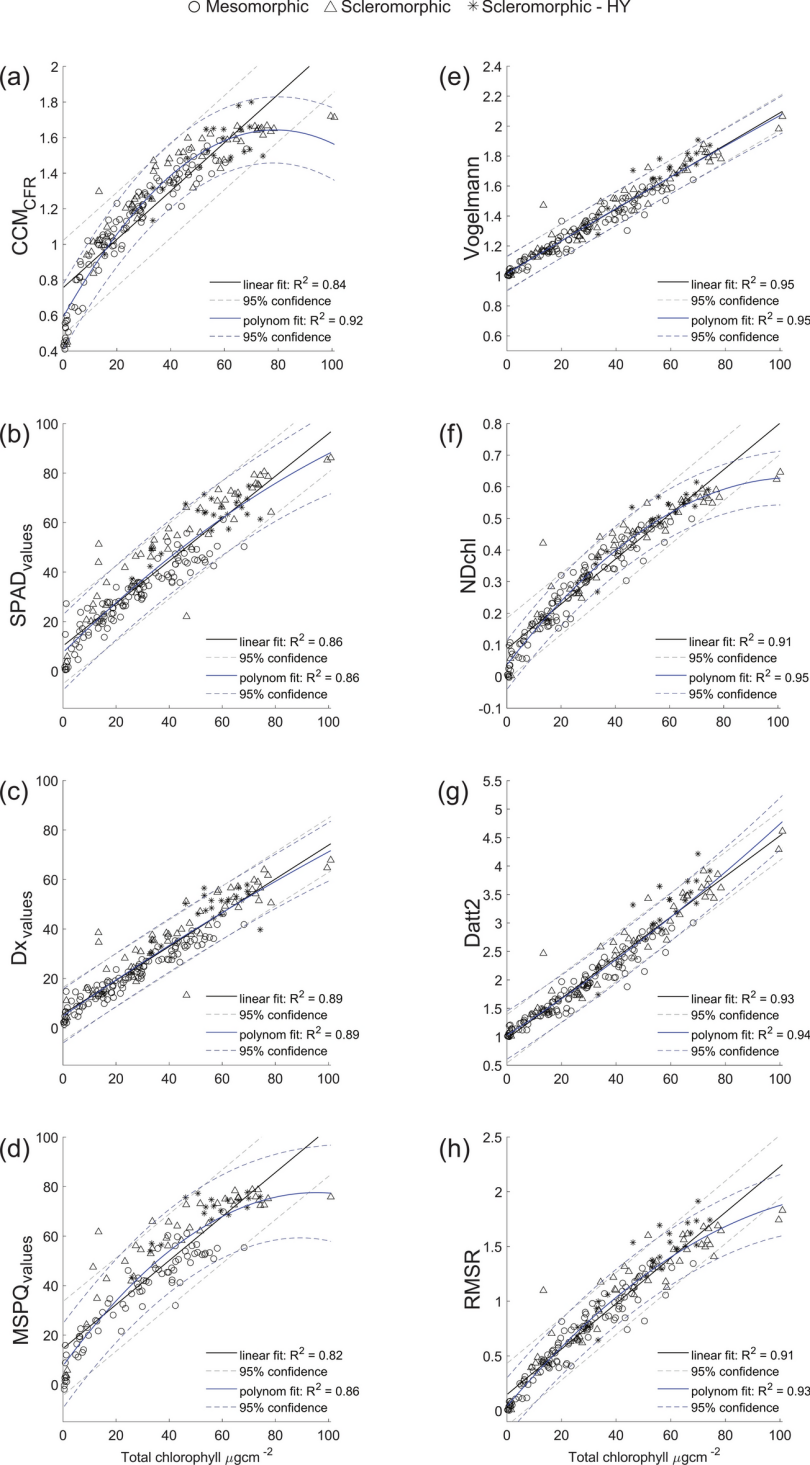
The best performing and linear relationships between Chl assessed spectrophotometrically in the lab (Total chlorophyll µg cm^−2^) and measured with chlorophyll meter for the four tested chlorophyll meters for laminar leaves: CCM_values_ (**a**); SPAD_values_ (**b**); Dx_values_ (**c**); MSPQ_values_ (**d**). The relationships between Chl extracted in the lab (Total chlorophyll µg cm^-2^) and selected vegetation indices derived from contact probe spectra of laminar leaves: Vogelmann (**e**); NDchl (**f**); Datt (**g**); RMSR (**h**). Blue line corresponds to best fit (polynomial) models, black line corresponds to linear model. Dashed lines of respective colours correspond to 95% confidence intervals. Laminar leaves are symbol-coded according to the internal structure: circle for mesomorphic leaves, triangle for scleromorphic and star for scleromorphic—HY leaves.

The visualizations of the best and linear model fit between Chl_abs_ and Chl_opt_ are shown for laminar leaves (Fig. 4a–d). The equations for best fitting and linear models are presented in Supplementary table S6. For CCM_values_, Dx_values_, MSPQ_values_, SPAD_values_ the performance of polynomial regression was the best in comparison with linear and logarithmic models, showing very high R^2^ = 0.92, R^2^ = 0.89, R^2^ = 0.86, and R^2^ = 0.86, respectively. The nRMSE was also lowest for the polynomial models (6.6%, 8.9%, 8.2% and 10.5%, respectively). However, the nRMSE for linear fits was not much higher (9.6%, 9.0%, 8.2% and 11.7%, respectively; Supplementary table S6).

For laminar leaves, separate models were constructed for three leaf categories based on their internal structure: (1) mesomorphic leaves, which lack specialized anatomical adaptations, have a single-layer epidermis, and exhibit lower LMA; (2) scleromorphic leaves, adapted to drier environments, with a thicker cuticle and higher LMA; and (3) scleromorphic leaves with a hypodermis (scleromorphic-HY), represented by three *Ficus* species (Fig. 1). For mesomorphic leaves, the linear relation of Chl_abs_ and Chl_opt_ gained a high R^2^ (0.82–0.88) with nRMSE up to 11.7%. In scleromorphic leaves, the linear model performance was slightly weaker, (R^2^ = 0.73–0.84) for all portable chlorophyll meters readings with nRMSE up to 12.7%. In contrast, the linear models for Chl_abs_ and Chl_opt_ acquired by all chlorophyll meters had the lowest R^2^ (0.70–0.72) and the highest nRMSE (up to 15.4%) for scleromorphic-HY leaves (Supplementary table S4). Regarding VIs acquired from the laminar leaf reflectance spectra, the best model fit was achieved for Chl_abs_ and Chl_opt_ and polynomial regression of Chl_abs_: Vogelmann and NDchl (R^2^ = 0.95), Datt2 (R^2^ = 0.94) and RMSR (R^2^ = 0.93) for laminar leaves (Fig. 4e–h). The equations for the best fitting and linear models for laminar leaves chlorophyll content and VIs shown in Fig. 4 are provided in Supplementary table S7.

### Within laminar leaves, Chl_opt_ was independent of leaf internal structure

**Fig. 5:**
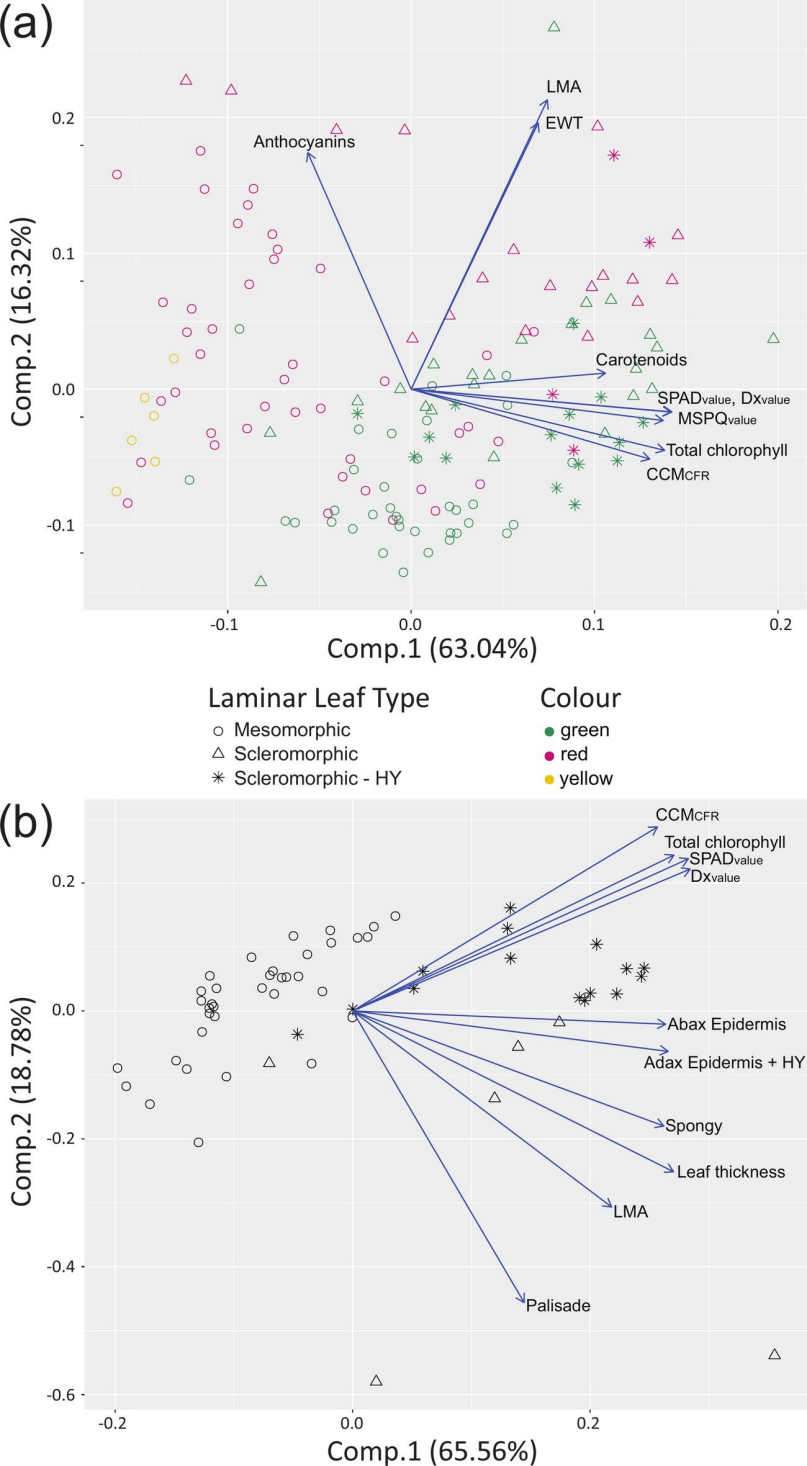
Principal component analysis for biophysical and anatomical traits of laminar leaves. Symbols correspond to scores of all samples within three laminar leaf categories based on leaf anatomy (circle = mesomorphic, triangle = scleromorphic, star = scleromorphic—HY). (**a**) Arrows show loadings of optically assessed chlorophyll: SPAD_value_, Dx_value_, MSPQ_value_, CCM_CFR_ and laboratory assessed biophysical traits Anthocyanins, Carotenoids, Total chlorophyll, EWT, and LMA. Colour categories correspond to pigment values and ratios: green = leaves with anthocyanin content below 20 nmol cm^2^, red = leaves with anthocyanin above 20 nmol cm^2^ and yellow = leaves with carotenoids to chlorophyll ratio values above 0.77; n = 143. (**b**) Arrows show loadings of anatomical traits: Leaf thickness, palisade parenchyma—Palisade, spongy parenchyma—Spongy, adaxial epidermis including hypodermis—Adax Epidermis + HY, abaxial epidermis—Abax Epidermis; biophysical traits Total chlorophyll, leaf mass per area—LMA; optically assessed chlorophyll: SPAD_value_, Dx_value_, CCM_CFR_; n = 55.

The PCA was applied to biophysical traits, biochemically and optically assessed pigment contents for laminar leaves (Fig. 5a). All values of Chl content Chl_abs_, Chl_opt_, and other pigments (Car_abs_, Anth_abs_) and biophysical traits such as LMA and EWT were included. Three laminar leaf anatomy subtypes (mesomorphic, scleromorphic, and scleromorphic-HY) were used as categorical variables and shape-coded in the PCA biplot. The descriptive statistics for LMA, EWT, and pigment contents are presented in Supplementary table S8.

The first principal component (PC1) explained 63% of total variability and the second component (PC2) explained 16% of variability in laminar leaf biophysical properties and chlorophyll meter readings. The first two components together described 79% of the variability in laminar leaf biophysical traits and chlorophyll meter readings. The PC1 included mainly Chl_abs_, Chl_opt_, and Car_abs_. Thus, PC1 reflected a positive contribution from photosynthetic pigment content, detected either biochemically or optically. PC2 was driven by Anth_abs_ and biophysical traits (LMA, EWT). The green leaves formed a cluster towards higher Chl content, while red leaves, with higher anthocyanin content, are widely spread across the PC1 and PC2 coordinate space, representing leaves from various phenophases with a wide range of anthocyanin and Chl content. Yellow leaves cluster separately, showing signs of senescence and Chl decay. Detailed anatomical analysis of tissue thickness was conducted on a subset of laminar leaf samples and descriptive statistics are shown for three leaf subgroups in Supplementary table S9.

The PCA was applied to anatomical traits, LMA, and Chl content (Chl_abs_, and Chl_op_). Three categories of laminar leaves (mesomorphic, scleromorphic, and scleromorphic- HY) were used as categorical variables and shape-coded in PCA biplot (Fig. 5b). The PC1 explained 66% and the PC2 19% of the total variability. Together PC1 and PC2 accounted for 87% of the variability in laminar leaf anatomical traits and Chl content. PC1 was primarily driven by epidermal thickness. PC2 contributed to distinguishing scleromorphic leaves with and without a hypodermis. Other anatomical traits (palisade parenchyma thickness, spongy parenchyma thickness, and leaf thickness) contributed to both PCAs, showing varying degrees of positive correlation with LMA. Surprisingly, palisade parenchyma thickness was largely independent of leaf Chl content. LMA and leaf thickness were key variables that helped separate mesomorphic leaves from scleromorphic ones.

### Independent validation of optical-absolute chlorophyll content models

**Fig. 6:**
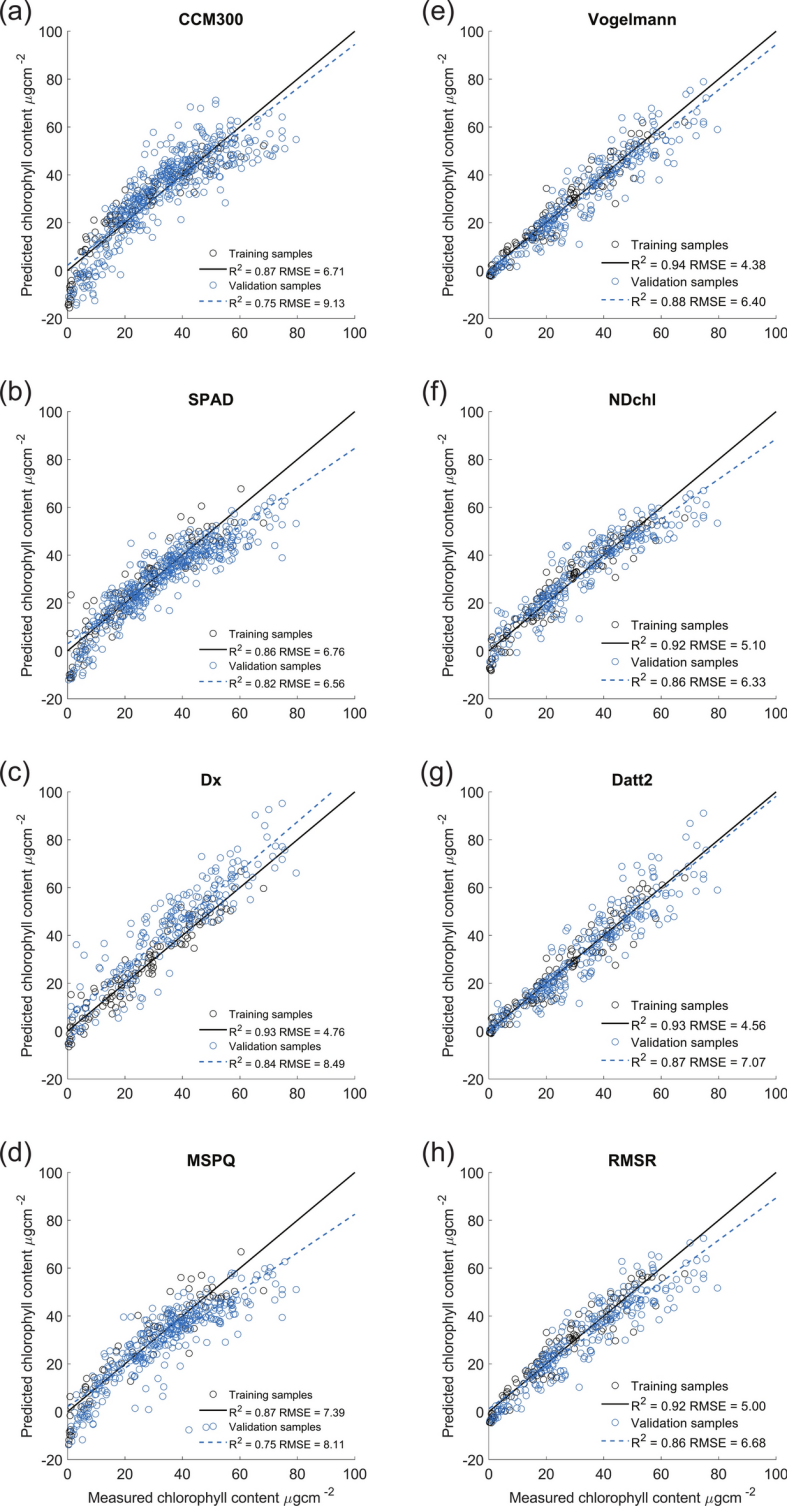
Visualization of the independent validation of linear models for Chl content prediction in laminar leaves. Validation performed on an independent dataset from seven deciduous tree species from floodplain forest. Predicted Chl contents were calculated from chlorophyll meter readings using linear model inversion. CCM_values_ (**a**); SPAD_values_ (**b**); Dx_values_ (**c**); MSPQ_values_ (**d**) and from VIs derived from contact probe measurements from leaf adaxial side using linear model inversion. Vogelmann (**e**); NDchl (**f**); Datt2 (**g**); RMSR (**h**). Black line corresponds training of the model on samples from the Charles University Botanical Garden, blue dashed line corresponds to the validation performed on data from Lanžhot floodplain forest.

All equations for the relationships between Chl_abs_ and Chl_opt_ for laminar leaves were inverted, and leaf Chl content was predicted from chlorophyll meter readings and VIs acquired from an independent dataset in a floodplain forest. Inversions of linear equations used for total chlorophyll content calculation of validation samples are presented in Supplementary table S10 for chlorophyll meters and Supplementary table S11 for VIs. The RMSE between predicted and biochemically assessed Chl content for each Chl prediction is presented in Supplementary table S12. In all cases, Chl values predicted by the inverted linear models showed the lowest RMSE in comparison with other model types (logarithmic and polynomial). The fit of predicted to measured Chl values of respective model training and independent validations are presented in Fig. 6 for all chlorophyll meters and VIs.

The developed linear models failed at predicting low Chl content (below 10 ug cm^-2^), producing negative Chl values (Fig. 6). The range of training datasets on laminar leaves collected in the Charles University Botanical Garden (Prague, Czech Republic) fully covered the observed ranges of Chl_abs_ and Chl_opt_ of the validation dataset from the floodplain forest (Fig. 6, Supplementary table S13). Validation of logarithmic models exhibited extremely high RMSE. The exponential model inversion resulted in severe outliers retrieved from higher index or chlorophyll meter readings. The inversion of polynomial models restricted Chl content prediction from higher values of VIs and chlorophyll meter readings (due to negative discriminant). In the case of predictions from VIs (NDchl and RMSR), the number of invalid values was minimal (up to six). However, for CCM300, the inversion of polynomial model resulted in high ratio of invalid predictions, and thus the RMSE was not calculated (Supplementary table S12). To sum up, the linear models of the relationships between Chl_abs_ and Chl_opt_ for laminar leaves were the most suitable (compared to polynomial and logarithmic models) showing excellent performance according to R^2^ and nRMSE up to 9,5% (Supplementary Table S4). Additionally, their advantage is that they can be easily inverted and validated with independent datasets^59^.

## Discussion

Rapid and non-destructive optical methods for assessing Chl content, allowing for the collection of a much larger number samples compared to laboratory biochemical determination, are increasingly being used in agriculture^60–62^, environmental science^63–65^, ecophysiology^66,67^ and remote sensing^9,68^. The relationship between biochemically and optically assessed Chl content is known to be heavily influenced by the Chl content itself^35^, measurement timing^62^, mineral nutrient availability^69^ and light conditions^49^. This study explored how leaf anatomy influences the reliability of optically versus biochemically measured Chl content. Additionally, both transmittance-based and fluorescence-based devices were evaluated.

The anatomy of leaves, rather than the studied species’ phylogenetic relationships, was the determining factor and source of uncertainty in the relationship between Chl_abs_ and Chl_opt_. This was reflected in the clustering of samples by species in grass (all from *Poaceae* family), in contrast to laminar leaves (12 different families). Parry et al.^30^ exhibited no significant difference in transmittance-based Chl_opt_ and Chl_abs_ relationship between monocot and dicot species (n = 5 and 17, respectively). By contrast, Cerovic et al.^32^ reported model improvement if monocot and dicot leaves were treated separately, which the authors attributed to the differences in leaf anatomy without empirical testing. Dong et al.^54^ inferred that Chl heterogeneous distribution within the leaf affects chlorophyll meter readings more than leaf structure. However, the study was conducted only on two monocot (corn and wheat) and two dicot (soybean and canola) crops^54^.

Brown et al.^31^ demonstrated that species-specific calibration functions decrease the nRMSE only for 2–5% in comparison to a generic function in selection of temperate deciduous species. In contrast, constructing separate regression models of Chl_abs_ and Chl_opt_ for individual grass species did not improve the weak relationship for all species together (Supplementary table S5). Our previous study on the same four species of wild grasses also pointed out difficulties in optically detected Chl at several hierarchical levels^70^. High uncertainty in Chl optical assessment by SPAD in mixed grasslands was also highlighted by Ludwig et al.^71^, who attributed the SPAD failure to specific leaf epidermal structures, such as dense hairs or waxes, contributing to specular reflection^72^. The limitation of using portable devices for Chl assessment in wild grasslands is a significant hurdle, as Chl is a crucial indicator of changes in leaf phenology and N content which are influenced by climate change and management practices^6^.

The striking difference between the relationship of SPAD_values_ and Chl_abs_ for laminar and grass leaves (Fig. 3b) may be due to the effect of leaf structure on leaf transmittance. Leaf transmittance is influenced by the venation density and bundle sheath distribution^50^, which in turn affects Chl_opt_ measurements by transmittance-based chlorophyll meters. Monocots typically have long, narrow leaves with parallel veins, such as grasses and palms. Dicots, usually have broader leaves with a network of veins, such as most trees and shrubs. These morphological and anatomical differences can affect how light interacts with the leaves and, consequently, the accuracy of chlorophyll measurements. As discussed by Cerovic^32^, there is a higher proportion of vascular tissue per unit surface area in monocots compared to dicots, which increases leaf transmittance. Due to the fluorescence rather than transmittance principle of measurement in CCM-300, this device was designed to measure Chl content on narrow leaves, needles, or other difficult samples^73^. Thus, we expected that regression models of Chl_abs_ and Chl_opt_ would improve for grass leaves in the case of using CCM and reach the model quality for laminar leaves. Unexpectedly, Chl_abs_ and Chl_opt_ exhibited high variance, frequently exceeding the 95% confidence interval of the regression model, particularly for *Deschampsia cespitosa* and *Nardus stricta* (Fig. 3d). These species possess unique leaf structures (Fig. 1), potentially deflecting excitation light from the chlorophyll meter. Besides deflecting the excitation light, other factors could affect chlorophyll meter readings in grass leaves, such as variations in leaf surface properties (e.g., waxy coatings, trichomes), presence of non-photosynthetic tissues (venation, sclerenchyma), and the presence of other pigments. These factors can alter the path and intensity of light within the leaf, affecting the readings from both transmittance and fluorescence-based chlorophyll meters. Observed performance of CCM-300 for measurements of narrow leaf samples was far beyond its nominal specification (R^2^ > 0.95) declared by the manufacturer.

To our knowledge, few studies have used optical Chl detection by CCM-300 on conifer needles^67,74–77^. Cited studies either applied the original Gittelson´s linear calibration trained on broadleaved species with dorsiventral laminar leaf^35^ or did not explicitly refer to any calibration. Based on weak relationship between Chl_abs_ and CCM_CFR_ on Norway spruce in this study (and also our unpublished results on silver fir and Scots pine), we strongly discourage taking CCM300 readings as the only ground truth for conifer species. Although the high uncertainty in chlorophyll assessment by portable meters presents a challenge, it is crucial to develop and refine methods that include conifers in ecophysiological analyses. Understanding the functional traits of conifers is essential for comprehensive studies of boreal forests and their ecosystem functions.

Due to the limitations in measuring optical properties, particularly transmittance caused by light scattering on needle edges^78^, transmittance-based portable meters were not tested on needles. In laboratory spectroscopy, special custom-made holders are used for measuring needle transmittance^79–81^. Considering the recent accessibility of 3D print, some adaptors for FOV reduction for a single needle measurement could be designed and tested with transmittance-based chlorophyll meters. However, similar sources of uncertainty, as seen in grass leaves, may arise due to the presence of a central cylinder with vascular bundles and other non-photosynthetic tissues (Fig. 1). Another relatively quick alternative to laboratory Chl assessment may be using the contact probe reflectance measurement of needles either attached^82,83^ to the shoot or detached from it^84,85^. Similarly to laminar leaves in our study, proper model training and validation on an independent dataset are necessary, as contact probe reflectance measurements of the needle layer introduce another source of uncertainty.

Two chlorophyll meters in our study (Dx and SPAD) were previously compared by others^32,54^, both finding Dx to have stronger correlation with biochemically determined Chl content than SPAD. Compared to Dong^54^, our Dx_values_ and SPAD_values_ closely correlated with each other (Fig. 5), and linear their models explained 86% and 89% variability and had comparable nRMSE (8.2% and 8.9%, respectively), thus, we cannot confirm the better estimation of Chl content by Dx than by SPAD. Dong et al.^54^ attributed the weaker SPAD-Chl_abs_ relationship to saturation caused by high leaf Chl content. Gitelson^35^ observed that leaf absorption at 680 nm and 750 nm remains stable once chlorophyll content exceeds 20 µg cm^-2^, and leaf absorption at 700 nm and 730 nm increases slowly for Chl content above 40 µg cm^-2^. This non-linear behaviour of leaf absorption may limit transmittance-based Chl quantification in Chl rich leaves. However, our study on woody species with laminar leaves did not show obvious saturation with high Chl content. Only 14% of our samples (28 out of 193) had a Chl content above 60 µg cm^-2^, with just two exceeding 80 µg cm^-2^ (both scleromorphic *Hedera helix* samples).

Scleromorphic samples clustered in space with positive values of PC1 and PC2, corresponding with higher LMA, EWT, and Chl content (Fig. 5). Moreover, the scleromorphic leaves with hypodermis formed a distinctive cluster. The location of scleromorphic scores in the PCA confirmed that leaf transmittance depends not only on Chl content but also on its distribution within leaves^49^, which is related to the arrangement of leaf tissues^52^. The Chl content in laminar leaves was not closely dependent either leaf thickness or palisade and spongy parenchyma thickness (Fig. 5b). Our results are in contrast with Neuwirthová et al.^86^, who presented a linear relationship (R^2^ = 0.43) between palisade parenchyma thickness and Chl a + b content in hemiboreal deciduous trees with mesomorphic leaves. The independence of chlorophyll meter readings on mesophyll thickness in the PCA (Fig. 5b) suggests that the linear relationship between Chl content and mesophyll tissue thickness is true only for a particular leaf anatomy, e.g., mesomorphic. Probably the detour effect may modulate the relationship between tissue thickness and Chl content in scleromorphic leaves and leaves with a hypodermis. Previous studies have shown that leaf Chl content can be quantitatively described by a quadratic function across a wide spectrum of leaf structures and vertical Chl distribution within the mesophyll^87^.

Radiative transfer modelling (RTM) at the leaf level has the potential to improve our understanding of how leaf internal structure affects optical properties and their relation to leaf biophysical traits, including Chl content and distribution. PROSPECT^88^, the most frequently used RTM does not account for asymmetry and variability in the structure of internal leaf tissues. At least two modified PROSPECT versions, FASPECT^89^ and INSPECT^90^ take into consideration leaf dorsiventrality, i.e., the difference between the adaxial and abaxial epidermis and palisade and spongy mesophyll. The most complex approach is represented by a 3D RTM that combines Monte Carlo ray tracing with a nearly realistic leaf mock-up consisting of vacuolized cells with chloroplasts^91,92^. However, all the mentioned RTMs account for the entire mesophyll profile, composed of either cells or airspaces, but do not include non-photosynthetic tissues such as vascular bundles and sclerenchyma. Considering recent imaging and computation technologies, such as 3D micro-computed tomography and machine learning^93^, which can provide reconstructions of internal leaf structure^94^, there is potential to modify existing RTMs to account for non-photosynthetic structures within the mesophyll.

Many studies have previously tested and compared the relationship between absolute Chl content and handheld chlorophyll meters, with several efforts to construct general and species-specific calibration equations published (see the references above). However, few studies have conducted independent validation to verify model performance. The successful validation was performed for relation of fluorescence ratio in 735 to 700 nm and total Chl content (principle of CCM-300 fluorescence based chlorophyll meter) on different species than those on which the model was trained^35^. In the case of transmittance-based chlorophyll meters, the comparison of an original dataset with previously derived Chl_abs_-Chl_opt_ relationships can be considered independent validations^30,32^. The case-specific relationships between Chl_abs_ and Chl_opt_ are frequently discussed as a downside of reflectance VIs^36^. Therefore, we focused on validation of Chl_abs_ and Chl_opt_ models trained on laminar leaves (Fig. 6) and confirmed excellent performance for all four tested chlorophyll meters and selected VIs. The RMSE in model validations increased only slightly in comparison to model training and the difference in RMSE was up to 3 µg of Chl per cm^2^. In general, the RMSE of all models trained (max 7.39 µg cm^2^ for MSPQ) and validated (max 9.13 µg cm^2^ for MSPQ) for laminar leaves showed reasonable values, comparable with the study by Casa et al.^95^ on crop species.

There are plenty of full-spectrum methods of Chl retrieval that may be less impacted by leaf internal structure and less prone to saturation than two band VIs, e.g., linear nonparametric methods as partial least squares regression (PLSR) or other machine learning algorithms^36^. Although we previously trained PLSR with contact probe spectra from the same laminar leaves (R^2^ = 0.95) in our earlier study^96^, this study focuses on comparing simple VIs, which align with the principles used in portable meters, that utilize index and reference bands for measurements.

A quantitative evaluation of bias in canopy Chl content assessment using imaging spectroscopy, when ground-truth data are obtained solely with portable chlorophyll meters, is necessary to understand the trade-off between speed, feasibility, and accuracy in chlorophyll ground-truth sampling. The requirement for separate models for each species suggests that species-specific anatomical traits significantly influence in situ chlorophyll measurements. While single-species models can provide accurate predictions for specific species, they limit the generalizability of the results. This implies that for accurate chlorophyll assessment, calibration specific to the species under study is necessary, which could complicate large-scale or multi-species studies. Future research should focus on developing more robust models that account for species-specific variations while maintaining broader applicability.

### Conclusions

To conclude, leaf anatomy is mirrored in the biophysical trait LMA, which increases from laminar leaves to needles. In accordance with our first hypothesis H1, the study has demonstrated that a generic model (i.e., one that can predict chlorophyll content across multiple species and leaf anatomical types) for the relationship between laboratory-determined Chl content (Chl_abs_) and chlorophyll meter readings (Chl_opt_) is not satisfactory for such heterogeneous anatomies, regardless of the measurement principle used by the chlorophyll meter (transmittance or fluorescence). In contrast to hypothesis H2, all four tested chlorophyll meters (CCM-300, SPAD, Dualex and MultispeQ) performed very well (R^2^ > 0.80) in laminar leaves, regardless the variance in their anatomy (e.g., thin dorsiventral mesomorphic leaves, thicker scleromorphic leaves, and scleromorphic leaves with hypodermis). Similarly, the four VIs (Vogelmann, NDchl, Datt2, and RMSR) were satisfactory predictors of Chl content in laminar leaves. The linear models for laminar leaves were successfully validated on an independent dataset with equally good performance for all tested chlorophyll meters and VIs. We infer that all tested devices can be used for taking Chl content ground truth without constraints in laminar leaves (confirmation of the hypothesis H3).

Further, we conclude that for grasses (and monocot crops) with wider leaves, such as *Calamagrostis villosa* and *Molinia caerulea,* the transmittance-based chlorophyll meters are viable (in contrast to grasses with narrower leaves and specific structure as *Deschampsia cespitosa)*. We recommend performing a calibration with Chl biochemical assessment for wild grass species with specific leaf structure under study before using only chlorophyll meter readings as a ground truth. The relationship of Chl_abs_ and Chl_opt_ was weak for Norway spruce needles (R^2^ = 0.45). There is a lack of studies available for validation of fluorescence-based chlorophyll meters on gymnosperm needles, thus taking Chl content ground truth without biochemical assay in conifers may produce unreliable results.

Our study demonstrates that while portable chlorophyll meters perform well for laminar leaves and grasses with wider leaves, their accuracy is limited for conifer needles and narrow grass leaves. Species-specific calibrations are necessary to account for anatomical variations, and adjustments in sampling protocols may be required to improve measurement reliability. These findings highlight the importance of considering leaf anatomy in optical chlorophyll assessment and call for the development of more robust models and techniques that can accurately measure chlorophyll content across diverse plant species and leaf structures.

## Material and methods

In this study we investigated three distinctive leaf anatomy: laminar (i.e., deciduous woody species) leaves, grass leaves, and needles (Fig. 1). The three groups are defined as follows: (1) Laminar leaves—dorsiventrally flattened (bifacial) leaves of woody angiosperms^43^ with differentiated palisade and spongy mesophyll layers and reticulate anastomosing vasculature. We further distinguished three anatomical subtypes of laminar leaves (1a) mesomorphic leaves of deciduous tree species, (1b) scleromorphic leaves of evergreen trees and shrubs, and (1c) scleromorphic leaves with pronounced hypodermis represented by *Ficus* species. (2) Grass leaves—C3 grasses with bifacial strap-like leaves possessing undifferentiated mesophyll and longitudinally arranged vasculature. And (3) Needles—equilateral gymnosperm leaves without differentiated mesophyll and vascular bundle in the central cylinder represented by Norway spruce (*Picea abies*) with irradiance induced differentiation into sun and shaded ecotypes. Chlorophyll content and the presence of other pigments, such as anthocyanins, also influenced leaf colour. In this study, leaves of different colours (green, red, and yellow) were chosen to cover a wider range of pigment composition and ratios present across the leaf development.

### Collection of leaf samples

Leaves were collected at four different locations in the Czech Republic during the growing seasons 2019, 2020, and 2021. The dates and locations of the collections by leaf anatomical categories (laminar leaves, grass leaves, needles) are shown in Supplementary table S14 and Supplementary figure S15). Woody plants with laminar bifacial leaves with differentiated mesophyll were collected in the Botanical Garden of Charles University in Prague (50.072N, 14.424E). Plants were selected according to leaf anatomy characterized by one of the following subtypes: (1) mesomorphic, (2) scleromorphic and (3) scleromorphic with hypodermis; the list of species in each group and their assignment to taxonomical units (families), including the database number are presented in Supplementary table S16. The species identity was confirmed by the botanist from the Botanical Garden of Charles University Science Faculty, Mgr. Tomáš Procházka. Shaded leaves were typically sampled from the ground.

For independent verification of the relationship between Chl content to chlorophyll meter reading, leaves were sampled in the floodplain forest at the confluence of the rivers Morava and Dyje, near the town of Lanžhot (48.682N, 16.946E) using deciduous woody plants with laminar bifacial leaves and differentiated mesophyll. Sunlit and shaded branches were cut by a tree climber from mature trees of *Acer campestre* L., *Carpinus betulus* L., *Fraxinus angustifolia* Vahl., *Populus alba* L., *Quercus cerris* L., *Quercus robur* L. and *Tilia cordata* Mill. For more details of see^97^.

Grass leaves were represented by four coexisting wild species from *Poaceae* family (*Calamagrostis villosa* (Chaix) J.F.Gmel., *Deschampsia cespitosa* (L.) P.Beauv., *Molinia caerulea* (L.) Moench and *Nardus stricta* L.). The species’ identities were confirmed by the botanist from the Krkonoše Mountains National Park Administration. Leaves were collected in relict alpine-arctic grass tundra in the Giant (Krkonoše) Mountains (50.734N, 15.696E). For each species, six plots with homogeneous canopy cover of the species were sampled–for more details see^98^.

Needle leaves were represented by mature trees of Norway spruce (*Picea abies* (L.) H. Karst.) collected at the experimental station Bílý Kříž, Beskydy Mountains, Czech Republic (49.503N, 18.539E). Sunlit and shaded branches were cut by a tree climber, and samples were taken from the current year’s needles, the previous year’s needles, and four-year-old needles. For more details see^99^.

### Leaf sampling

Laminar leaves: leaves were measured immediately after being detached from the branch or stored in a refrigerator for no more than 30 min before processing. First, the reflectance of the leaves was measured using a spectroradiometer and a contact probe. Second, all portable chlorophyll meters were used and (Chl_opt_) was measured. Third, one disk (area = 68 mm^2^) was cut from each leaf for Chl (Chl_abs_) and anthocyanin extraction. Finally, a square segment of the leaf was cut out and immersed in fixative solution for anatomical analysis. A second leaf of similar size, colour, position in the canopy, and developmental stage was removed from the branch, weighed, scanned, and later dried and weighed again. This “twin” was used to assess LMA, EWT.

Grass leaves: chlorophyll meter readings were taken on grass leaves attached to the plant using a chlorophyll meter (CCM_values_,) then leaves were collected immediately in the field for Chl extraction (Chl_abs_). A 2 cm long leaf segment was cut, flattened under a microscope glass, photographed for area assessment, and stored in plastic vials in a refrigerator before freezing. A subsample was weighed fresh, scanned, and dried for calculation of LMA and EWT.

Needles: Shoots were separated from the branch, sorted by age, and stored in a refrigerator for no longer than 24 h before processing. First, CCM_values_ were taken from the middle part of three needles and the same needles were used for Chl extraction (Chl_abs_). A second parallel set of needles was immersed in fixative solution for anatomical analysis. The third set of needles was weighed fresh, scanned, and dried for calculation of LMA and EWT.

### Optical assessment of Chl content using portable chlorophyll meters

Three transmittance-based chlorophyll meters: SPAD-502 (SPAD), Dualex-4 Scientific (Dx) and MultispeQ (MSPQ), and one fluorescence chlorophyll meter: CCM-300 (CCM), were used for optical assessment of Chl content in leaves (Chl_opt_, Table 1). For laminar leaves, three readings were taken on each leaf with each instrument from the adaxial leaf side. Measurements were taken in the central part of the leaf, avoiding the midrib and main veins. The three measurements were averaged, and the average was used as a representative value for the leaf. Measurements with all four chlorophyll meters (SPAD_values_, Dx_values_, MSPQ_values_, CCM_values_) were obtained for laminar leaves. For grass leaves, Chl values were measured at a single location in the apical third of the leaf blade. All four grass species were measured by CCM (CCM_values_). A subset of CCM-300 readings on grass species was already published in Červená et al.^70^ as a ground truth for chlorophyll content mapping from aerial hyperspectral data. Three species with a wide enough lamina to cover the SPAD measurement area (*Calamagrostis villosa*, *Deschampsia cespitosa*, and *Molinia careulea*) were also measured by SPAD and SPAD_values_ detected. For the spruce needles, three needles were measured only once with the CCM (CCM_values_), always taking a reading in the central part of the needle. The average of these three needle measurements was used to relate to Chl_abs_.

**Table 1:** The list of portable chlorophyll meters used in this study and their main technical characteristics. SPAD-502 (Konica–Minolta, Inc., Osaka, Japan); Dualex-4 Scientific (FORCE-A, Orsay, France); MultispeQ (PhotosynQ Inc., East Lansing, MI, USA); CCM-300 (OptiSciences, Inc., Hudson, NH, USA). The first three transmittance-based chlorophyll meters do require the leaf covering full measurement area in contrast to fluorescence-based CCM-300, which does not.

Chlorophyll meter (abbreviation)	Chl_opt_	Optical principle	Index band (nm)	Reference band (nm)	Units, range	Measurement area Shape, size	References
SPAD-502 (SPAD)	SPAD_values_	Transmittance	650	940	Relative, 0–50	Rectangular, 6 mm^2^	^53,100^
Dualex-4 Scientific (Dx)	Dx_values_	Transmittance	710	850	µg cm^−2^, 5–80	Circular, 20 mm^2^	^32^
MultispeQ V2.0 (MSPQ)	MSPQ_values_	Transmittance	650*	940*	Relative, 0–80	Square, 64 mm^2^	^29^
CCM-300 (CCM300)	CCM_values_ CCM_CFR_	Chlorophyll fluorescence	700	735	CFR** or mg m^−2^, 41–675	Circular, 7 mm^2^	^35,68^

*MSPQ uses a series of transmittance measurements over a range of progressively increasing light intensities to increase the dynamic range of the results, particularly with leaves that have high Chl content or are unusually thick.

**Chlrorophyll fluorescence ratio in the 735 and 700 nm.

### Reflectance measurements and spectral processing

Reflectance was measured for laminar leaves collected in the Botanical Garden of Charles University in Prague and deciduous trees from floodplain forest. Leaf reflectance from the adaxial side of the leaves was measured with an ASD FieldSpec 4 Wide-Res spectroradiometer with attached contact probe (ASD Inc., Boulder, CO, USA). Three measurements per leaf were always taken, when leaf size allowed. Measurements were placed at the same locations where chlorophyll meter readings were taken. Leaf reflectance spectra ranging from 350 to 2500 nm were normalized against a white reference spectrum (99% Spectralon white panel) to obtain relative reflectance spectra. The median of the spectral curve from three measurements was used as a representative value for the leaf. Four VIs (VI) Datt2, NDChl, RMSR and Vogelmann were selected based on our previous experience with very good linear relationship (R^2^ > 0.85) to Chl_abs_^86^. The VIs were calculated in R (4.1.2) (R Core Team, 2021) based on the literature presented in Supplemetary Table S17.

### Biochemical assessment of leaf pigments: chlorophylls, carotenoids, and anthocyanins

Photosynthetic pigments (chlorophyll a, b, and total carotenoids) were determined spectrophotometrically from DMF extracts. Pigment content was calculated according to^101^ and related to leaf area. Chl a and b were summed together and further expressed by the abbreviation Chl_abs_. Anthocyanins were extracted in acidified methanol according to^102^ and converted to molar concentration using the Beer-Lambert equation with the universal molar extinction coefficient ε = 30,000 l mol^−1^ cm^−1^^27^ and related to leaf area. Biochemically detected anthocyanins are hereafter referred to as Anth_abs_.

Biochemically determined leaf chlorophyll contents of grass leaves have been published previously^98^ for the purpose of species competition strategies but not in relation to chlorophyll meter readings. The biochemically determined chlorophyll contents for laminar leaves from floodplain forest have been published previously^97^ as ground truth for satellite observations but not in relation to chlorophyll meter readings. The biochemically determined chlorophyll contents for laminar leaves from Charles University Botanical Garden have been published previously in Neuwirthová et al.^96^ context of PLSR modelling but not in relation to chlorophyll meter readings.

### Biophysical and anatomical analyses

Leaf mass per area (LMA, mg cm^-2^) was determined by first scanning laminar and grass leaves using an EPSON Perfection V600 Photo scanner with 300 dpi resolution and calculating leaf area using ImageJ and Matlab R2020b. The water content of the leaves was expressed as equivalent water thickness (EWT, mg cm^-2^), which was calculated from the fresh and dry weight difference and area of the scanned sample. The needles were scanned at 600 dpi resolution and their projected area was evaluated in Matlab R2020b. LMA per area measurement for spruce needles in based on the hemi-surface area of the needles, which was calculated using the conversion factors according to (Homolová et al., 2013), and further used to calculate the LMA and EWT. All leaves were dried for 72 h at 60 °C and weighed.

Laminar leaves: for anatomical analyses, a 5 × 5 mm segment, excluding the midrib and major veins, was fixed in 70% FAA (70% ethanol, formalin, acetic acid; 18:1:1, respectively) and stored for later sectioning. The sample was excised from one of the sites of chlorophyll meter measurements. Leaves were then cut using a hand-held microtome into sections approximately 80 μm thick, stained with toluidine blue or phloroglucinol, and photographed. Light microscope images (five per leaf) were taken using an Olympus BX40 microscope equipped with a Canon EOS100D camera (Canon Inc., Tokyo, Japan). Leaf thickness, mesophyll thickness, palisade, and spongy parenchyma thickness, and adaxial (including hypodermis) and abaxial epidermis thickness were measured using ImageJ software. Leaf tissue thickness measurements have been published previously^96^ in context of PLSR modelling but not in relation to chlorophyll meter readings.

Grass leaves: fresh leaves were cut using hand-held microtomes to a section thickness of about 80 μm, stained with phloroglucinol and photographed using an Olympus BX40 microscope equipped with a Canon EOS100D camera (Canon Inc., Tokyo, Japan).

Needles: fresh leaves were cut using hand-held microtomes to a section thickness of about 80 μm, stained with phloroglucinol, and photographed using an Olympus BX40 microscope equipped with a Canon EOS100D camera.

The area of photosynthetic (mesophyll) and non-photosynthetic (epidermis, vascular bundles, sclerenchyma, hypodermis) tissues was assessed using the point counting method on phloroglucinol-stained or fluorescence images. The ratio of mesophyll to non-photosynthetic tissues on a cross section was used for further comparison. Proportion of mesophyll, epidermis, vascular bundles, and sclerenchyma in grass leaves have been published previously^98^ for the purpose of species competition strategies but not in relation to chlorophyll meter readings.

### Statistical analyses

Statistical models for relationships between independent variables (Chl_abs_) and dependent variable (Chl_opt_) and four VIs include linear, logarithmic, and polynomial regression equations constructed in Matlab R2020b. For each regression model the R^2^, RMSE (root mean square error in given variable units) and RMSE normalized by the range (max–min) of the given value (nRMSE) was calculated. Usually, the best performing models were evaluated according to the highest value of coefficient of determination (R^2^) and the lowest nRMSE.

For independent validation of the statistical prediction of Chl_abs_ based on Chl_opt_, the model equations were inverted. The chlorophyll meter readings and VIs from samples collected in the Lanžhot floodplain forest were used as model inputs. The relationships of estimated and measured Chl values were evaluated according to the coefficients of determination and RMSE (root mean squared error).

All values of Chl content Chl_abs_, Chl_opt_, and other biochemical and biophysical traits (e.g., Car_abs_, Anth_abs_, LMA, EWT) were analysed by principal component analysis (PCA) to reveal intercorrelation of variables in laminar leaves. Relation of biophysical properties detected by biochemical and optical approach were determined in R (4.1.2)^103^ using the ggfortify package^104^.

## Supplementary Information

Supplementary Information.

## Data Availability

Data are available in Zenodo repository found by https://zenodo.org/records/14615430.
