# Supplementary material for: Mind the leaf anatomy while taking ground truth with portable chlorophyll meters
Source: Sci Rep. 2025 Jan 13;15:1855. doi: 10.1038/s41598-024-84052-5 (PMC11730753; doi:10.1038/s41598-024-84052-5)
Supplement: Supplementary file 1 — Supplementary Information. [file 41598_2024_84052_MOESM1_ESM.docx]

Mind the leaf anatomy while taking ground truth with portable chlorophyll meters.

Supplementary Materials

Zuzana Lhotáková^a*^, Eva Neuwirthová^a^, Markéta Potůčková^b^, Lucie Červená^b^, Lena Hunt^a^, Lucie Kupková^b^, Petr Lukeš^c^, Petya Campbell^d^, Jana Albrechtová^a^

^a^Department of Plant Experimental Biology, Faculty of Science, Charles University, Viničná 5, 12800, Prague, Czech Republic; [zuzana.lhotakova@natur.cuni.cz](mailto:zuzana.lhotakova@natur.cuni.cz), [eva.neuwirhtova@natur.cuni.cz](mailto:eva.neuwirhtova@natur.cuni.cz), [lenamhunt@gmail.com](mailto:lenamhunt@gmail.com), [jana.albrechtova@natur.cuni.cz](mailto:jana.albrechtova@natur.cuni.cz)

^b^Department of Applied Geoinformatics and Cartography, Faculty of Science, Charles University, Albertov 6, 12800, Prague, Czech Republic; [marketa.potuckova@natur.cuni.cz](mailto:marketa.potuckova@natur.cuni.cz) , [lucie.cervena@natur.cuni.cz](mailto:lucie.cervena@natur.cuni.cz) , [lucie.kupkova@natur.cuni.cz](mailto:lucie.kupkova@natur.cuni.cz)

^c^ Global Change Research Institute of the Czech Academy of Sciences, Bělidla 986/4a, 60300, Brno, Czech Republic; [lukes.p@czechglobe.cz](mailto:lukes.p@czechglobe.cz)

^d^University of Maryland Baltimore County and NASA/Goddard Space Flight Center, Code 618, Greenbelt, MD 20771 USA; [petya@umbc.edu](mailto:petya@umbc.edu)

***Author to whom correspondence should be addressed:** [zuzana.lhotakova@natur.cuni.cz](mailto:zuzana.lhotakova@natur.cuni.cz)

**Supplementary Table S1.** Chlorophyll and carotenoids contents, their ratio, and structural parameters (leaf mass per area, LMA and ratio of mesophyll to non-photosynthetic tissues on a leaf cross section for three examined anatomical groups: Laminar leaves, Grass leaves and Needles. The analysis was conducted only on a subset of samples due to labour demands of leaf sectioning.

|  |  | Chlorophyll µg.cm^-2^ | Carotenoids µg.cm^-2^ | Carotenoids to chlorophyll | Mesophyll to Non-photosynthetic tissues | LMA  mg.cm^-2^ |
| --- | --- | --- | --- | --- | --- | --- |
| Laminar leaves | mean | 35.33 | 5.12 | 0.33 | 1.64 | 5.90 |
|  | standard deviation | 19.46 | 1.86 | 0.58 | 0.64 | 2.97 |
|  | min | 1.19 | 2.89 | 0.09 | 0.60 | 2.58 |
|  | max | 69.98 | 9.10 | 2.42 | 3.20 | 14.92 |
|  | range | 68.79 | 6.21 | 2.33 | 2.60 | 12.34 |
|  | N | 16 | 16 | 16 | 16 | 16 |
| Grass leaves | mean | 38.05 | 5.34 | 0.15 | 1.24 | 9.62 |
|  | standard deviation | 12.76 | 1.41 | 0.05 | 0.43 | 2.33 |
|  | min | 18.97 | 2.18 | 0.08 | 0.60 | 6.16 |
|  | max | 59.17 | 7.82 | 0.32 | 2.10 | 14.35 |
|  | range | 40.20 | 5.64 | 0.24 | 1.50 | 8.19 |
|  | N | 24 | 24 | 24 | 23 | 24 |
| Needles | mean | 54.23 | 6.76 | 0.12 | 2.71 | 16.66 |
|  | standard deviation | 12.69 | 2.07 | 0.02 | 0.22 | 4.06 |
|  | min | 35.40 | 3.85 | 0.10 | 2.30 | 9.52 |
|  | max | 95.03 | 12.42 | 0.15 | 3.10 | 23.34 |
|  | range | 59.63 | 8.57 | 0.05 | 0.80 | 13.82 |
|  | N | 30 | 30 | 30 | 30 | 30 |

## Supplementary Table S2. Equations for the linear models for three leaf types according to their anatomy (Laminar leaves, Grass leaves and Needles) shown in Figure 3. Coefficients of determination (R^2^), root mean square error (RMSE) and RMSE normalized by the variable range (nRMSE in %)

| **CCM 300** | | | | | |
| --- | --- | --- | --- | --- | --- |
| **Leaf type** | **Linear model** | **R^2^** | **RMSE [CCM_CFR_ values]** | | **nRM**  **SE%** |
| Laminar | ${CCM}_{CFR}= 0.0136TC+0.7554$ | 0.84 | 0.13 | | 9.59 |
| Grass | ${CCM}_{CFR}= 0.0080TC+0.$8856 | 0.44 | 0.12 | | 11.50 |
| Needle | ${CCM}_{CFR}= 0.0041TC+0.8672$ | 0.30 | 0.11 | | 12.75 |
| All samples | ${CCM}_{CFR}= 0.0069TC+0.8877$ | 0.33 | 0.17 | | 11.95 |
| **SPAD** | | | | | |
| **Leaf type** | **Linear model** | **R^2^** | **RMSE [SPAD values]** | **nRM**  **SE%** | |
| Laminar | $SPAD= 0.8577TC+10.1521$ | 0.86 | 7.70 | 8.98 | |
| Grass | $SPAD= 0.1670TC+28.1166$ | 0.08 | 7.75 | 16.21 | |
| All samples | $SPAD= 0.5183TC+17.2672$ | 0.42 | 10.06 | 17.74 | |

## Supplementary table S3. Equations for the best fitting models for three leaf types according to their anatomy (Laminar leaves, Grass leaves and Needles) and all leaf types together for CCM300 and SPAD. TC – total chlorophyll in μg cm^-2^; the natural logarithm ln(x)

| **CCM 300** | | | | | |
| --- | --- | --- | --- | --- | --- |
| **Leaf type** | **Best fitting model** | **R^2^** | **RMSE [CCM_CFR_ values]** | **nRM**  **SE%** | |
| Laminar | ${CCM}_{CFR}= -0.0002{TC}^{2}+0.0267TC+0.5900$ | 0.92 | 0.09 | 6.63 | |
| Grass | ${CCM}_{CFR}= 0.1663+0.2863\ln\left( TC \right)$ | 0.45 | 0.12 | 11.43 | |
| Needle | ${CCM}_{CFR}= -0.0001{TC}^{2}+0.0136TC+0.6721$ | 0.45 | 0.10 | 11.33 | |
| All samples | ${CCM}_{CFR}= 0.4547+0.1993\ln\left( TC \right)$ | 0.43 | 0.15 | 11.02 | |
| **SPAD** | | | | | |
| **Leaf type** | **Best fitting model** | **R^2^** | **RMSE [SPAD values]** | | **nRM**  **SE%** |
| Laminar | $SPAD= -0.0026{TC}^{2}+1.0587TC+7.4766$ | 0.86 | 7.58 | | 8.85 |
| Grass | $SPAD= -0.0022{TC}^{2}+1.8718TC-1.2854$ | 0.32 | 6.68 | | 13.98 |
| All samples | $SPAD= -0.0023{TC}^{2}+0.6949TC+14.4365$ | 0.42 | 10.04 | | 11.71 |

**Supplementary Table S4.** Coefficients of determination (R^2^), root mean square error (RMSE) and RMSE normalized by the variable range (nRMSE in %) of all tested regressions for Chl_abs_ and Chl_opt_. POL – polynomial regression, LIN – linear regression, LOG – logarithmic regression, scleromorphic – HY – scleromorphic with pronounced hypodermis.

| **Laminar leaves, all types** | | | | | | | | | | | |  |  | **Laminar leaves, mesomorphic** | | | | | | | | | |
| --- | --- | --- | --- | --- | --- | --- | --- | --- | --- | --- | --- | --- | --- | --- | --- | --- | --- | --- | --- | --- | --- | --- | --- |
|  | POL |  |  | LIN | |  |  | LOG | |  |  |  |  |  | POL |  |  | LIN |  |  | LOG |  |  |
| Device/Index | R^2^ | RMS  E | nRM  SE% | | R^2^ | RMS  E | nRM  SE% | | R^2^ | RMS  E | nRM  SE% |  |  | Device/Index | R^2^ | RMSE | nRMSE% | R^2^ | RMSE | nRMSE% | R^2^ | RMSE | nRMSE% |
| CCM | 0.924 | 0.09 | 6.6 | 0.840 | | 0.13 | 9.6 | 0.88 | | 0.12 | 8.3 |  |  | CCM | 0.936 | 0.08 | 6.7 | 0.868 | 0.11 | 9.5 | 0.891 | 0.10 | 8.7 |
| SPAD | 0.863 | 7.59 | 8.9 | 0.859 | | 7.70 | 8.9 | 0.70 | | 11.16 | 13.0 |  |  | SPAD | 0.883 | 4.80 | 7.9 | 0.861 | 5.19 | 8.5 | 0.763 | 6.77 | 11.1 |
| Dx | 0.889 | 5.43 | 8.2 | 0.888 | | 5.44 | 8.2 | 0.64 | | 9.73 | 14.7 |  |  | Dx | 0.931 | 2.88 | 6.4 | 0.929 | 2.91 | 6.5 | 0.717 | 5.79 | 13.0 |
| MSPQ | 0.856 | 8.44 | 10.5 | 0.818 | | 9.48 | 11.7 | 0.78 | | 10.42 | 13.4 |  |  | MSPQ | 0.908 | 5.58 | 7.2 | 0.870 | 6.59 | 8.5 | 0.840 | 7.33 | 9.4 |
| Vogelmann | 0.947 | 0.06 | 5.4 | 0.947 | | 0.06 | 5.4 | 0.68 | | 0.14 | 13.2 |  |  | Vogelmann | 0.942 | 0.04 | 6.7 | 0.939 | 0.04 | 6.8 | 0.722 | 0.09 | 14.6 |
| NDchl | 0.946 | 0.04 | 6.0 | 0.914 | | 0.05 | 7.5 | 0.78 | | 0.08 | 11.9 |  |  | NDchl | 0.943 | 0.03 | 6.8 | 0.919 | 0.04 | 8.8 | 0.789 | 0.07 | 12.9 |
| Datt2 | 0.936 | 0.21 | 5.8 | 0.934 | | 0.21 | 5.9 | 0.62 | | 0.51 | 14.2 |  |  | Datt2 | 0.934 | 0.14 | 7.0 | 0.934 | 0.14 | 7.0 | 0.672 | 0.31 | 15.7 |
| RMSR | 0.930 | 0.13 | 6.8 | 0.914 | | 0.14 | 7.5 | 0.74 | | 0.25 | 13.1 |  |  | RMSR | 0.937 | 0.10 | 7.3 | 0.922 | 0.11 | 8.0 | 0.768 | 0.19 | 13.9 |
|  |  |  |  |  | |  |  |  | |  |  |  |  |  |  |  |  |  |  |  |  |  |  |
| **Laminar leaves, scleromorphic** | | | | | | | | | | | |  |  | **Laminar leaves, scleromorphic - HY** | | | | | | | | | |
|  | POL |  |  | LIN | |  |  | LOG | |  |  |  |  |  | POL |  |  | LIN |  |  | LOG |  |  |
| Device/Index | R^2^ | RMSE | nRMSE% | R^2^ | | RMSE | nRM  SE% | R^2^ | | RMSE | nRMSE% |  |  | Device/Index | R^2^ | RMSE | nRMSE% | R^2^ | RMSE | nRMSE% | R^2^ | RMSE | nRMSE% |
| CCM | 0.896 | 0.10 | 7.6 | 0.783 | | 0.14 | 10.9 | 0.930 | | 0.08 | 6.2 |  |  | CCM | 0.799 | 0.10 | 11.3 | 0.727 | 0.11 | 12.9 | 0.777 | 0.10 | 11.7 |
| SPAD | 0.854 | 8.10 | 9.8 | 0.837 | | 8.47 | 10.3 | 0.775 | | 9.97 | 12.1 |  |  | SPAD | 0.857 | 4.79 | 9.3 | 0.727 | 6.48 | 12.6 | 0.817 | 5.30 | 10.3 |
| Dx | 0.845 | 6.72 | 10.6 | 0.840 | | 6.77 | 10.6 | 0.694 | | 9.36 | 14.7 |  |  | Dx | 0.858 | 4.55 | 10.4 | 0.740 | 6.01 | 13.7 | 0.811 | 5.12 | 11.7 |
| MSPQ | 0.850 | 7.29 | 9.7 | 0.735 | | 9.56 | 12.7 | 0.881 | | 6.41 | 8.5 |  |  | MSPQ | 0.842 | 3.96 | 11.5 | 0.700 | 5.30 | 15.4 | 0.782 | 4.51 | 13.1 |
| Vogelmann | 0.941 | 0.06 | 6.1 | 0.940 | | 0.07 | 6.1 | 0.760 | | 0.13 | 12.4 |  |  | Vogelmann | 0.909 | 0.06 | 8.3 | 0.907 | 0.06 | 8.2 | 0.876 | 0.07 | 9.5 |
| NDchl | 0.925 | 0.04 | 6.9 | 0.878 | | 0.06 | 8.7 | 0.845 | | 0.06 | 9.8 |  |  | NDchl | 0.914 | 0.03 | 7.7 | 0.887 | 0.04 | 8.7 | 0.908 | 0.03 | 7.8 |
| Datt2 | 0.936 | 0.23 | 6.4 | 0.936 | | 0.23 | 6.4 | 0.721 | | 0.48 | 13.3 |  |  | Datt2 | 0.876 | 0.26 | 9.5 | 0.876 | 0.26 | 9.2 | 0.833 | 0.30 | 10.7 |
| RMSR | 0.908 | 0.15 | 8.0 | 0.879 | | 0.17 | 9.1 | 0.807 | | 0.21 | 11.5 |  |  | RMSR | 0.872 | 0.14 | 9.3 | 0.863 | 0.14 | 9.4 | 0.857 | 0.14 | 9.6 |

**Supplementary Table S4, continuing.** Coefficients of determination (R^2^), root mean square error (RMSE) and RMSE normalized by the variable range (nRMSE, in %) of all tested regressions for Chl_abs_ and Chl_opt_. POL – polynomial regression, LIN – linear regression, LOG – logarithmic regression.

| **Grass leaves, all species** | | | | | | | | | |  | **Grass leaves, *Calamagrostis villosa*** | | | | | | | | | | |
| --- | --- | --- | --- | --- | --- | --- | --- | --- | --- | --- | --- | --- | --- | --- | --- | --- | --- | --- | --- | --- | --- |
|  | POL |  |  | LIN |  |  | LOG |  |  |  |  | POL |  |  | LIN |  |  | LOG |  |  |  |
| Device | R^2^ | RMSE | nRMSE% | R^2^ | RMSE | nRMSE% | R^2^ | RMSE | nRMSE% |  | Device | R^2^ | RMSE | nRMSE% | R^2^ | RMSE | nRMSE% | R^2^ | RMSE | nRMSE% |  |
| CCM | 0.444 | 0.118 | 11.5 | 0.439 | 0.119 | 11.5 | 0.446 | 0.118 | 11.4 |  | CCM | 0.423 | 0.083 | 11.4 | 0.337 | 0.089 | 12.2 | 0.408 | 0.084 | 11.5 |  |
| SPAD | 0.314 | 6.681 | 14.0 | 0.077 | 7.741 | 16.2 | 0.134 | 7.498 | 15.7 |  | SPAD | 0.461 | 3.340 | 12.8 | 0.369 | 3.602 | 13.8 | 0.428 | 3.430 | 13.1 |  |
|  |  |  |  |  |  |  |  |  |  |  |  |  |  |  |  |  |  |  |  |  |  |
| **Grass leaves, *Deschampsia cespitosa*** | | | | | | | | | |  | **Grass leaves, *Molinia caerulea*** | | | | | | | | | | |
|  | POL |  |  | LIN |  |  | LOG |  |  |  |  | POL |  |  | LIN |  |  | LOG |  |  |  |
| Device | R^2^ | RMSE | nRMSE% | R^2^ | RMSE | nRMSE% | R^2^ | RMSE | nRMSE% |  | Device | R^2^ | RMSE | nRMSE% | R^2^ | RMSE | nRMSE% | R^2^ | RMSE | nRMSE% |  |
| CCM | 0.071 | 0.101 | 18.7 | 0.058 | 0.101 | 18.7 | 0.053 | 0.101 | 18.8 |  | CCM | 0.182 | 0.072 | 18.3 | 0.159 | 0.072 | 18.6 | 0.176 | 0.072 | 18.4 |  |
| SPAD | 0.060 | 2.783 | 17.7 | 0.059 | 2.775 | 17.7 | 0.057 | 2.777 | 17.7 |  | SPAD | 0.458 | 4.671 | 13.8 | 0.453 | 4.679 | 13.8 | 0.437 | 4.745 | 14.0 |  |
|  |  |  |  |  |  |  |  |  |  |  |  |  |  |  |  |  |  |  |  |  |  |
| **Grass leaves, *Nardus stricta*** | | | | | | | | | |  | **Needles, *Picea abies*** | | | | | | | | | | |
|  | POL |  |  | LIN |  |  | LOG |  |  |  |  | POL |  |  | LIN |  |  | LOG |  |  |  |
| Device | R^2^ | RMSE | nRMSE% | R^2^ | RMSE | nRMSE% | R^2^ | RMSE | nRMSE% |  | Device | R^2^ | RMSE | nRMSE% | R^2^ | RMSE | nRMSE% | R^2^ | RMSE | nRMSE% |  |
| CCM | 0.125 | 0.142 | 20.0 | 0.107 | 0.143 | 20.2 | 0.117 | 0.142 | 20.0 |  | CCM | 0.447 | 0.097 | 11.3 | 0.296 | 0.110 | 12,8 | 0.443 | 0.098 | 11.4 |  |

**Supplementary Table S5.** Equations for the best fitting and linear models for Grass leaves shown in **Figure 3**. TC – total chlorophyll in μg cm^-2^; the natural logarithm ln(x), coefficients of determination (R^2^), root mean square error (RMSE) and RMSE normalized by the variable range (nRMSE in %).

| **CCM 300** | | | | |
| --- | --- | --- | --- | --- |
| **Grass** | **Best fitting model**  **Linear model** | **R^2^** | **RMSE [CCM_CFR_ values]** | **nRMSE%** |
| All samples | ${CCM}_{CFR}= 0.1663+0.2863\ln\left( TC \right)$ | 0.45 | 0.118 | 11.4 |
| All samples | ${CCM}_{CFR}=$0.00799TC + 0.88557 | 0.44 | 0.118 | 11.5 |
| **SPAD** | | | | |
| **Grass** | **Best fitting model** | R2 | RMSE [SPAD values] | **nRMSE%** |
| All samples | $SPAD= -0.0217{TC}^{2}+1.8718TC-1.2854$ | 0.32 | 6.684 | 14.0 |
| All samples | $SPAD=$0.16702 TC + 28.11655 | 0.08 | 7.746 | 16.2 |
| *C.v.* + *M.c.* | $SPAD= -0.0050{TC}^{2}+1.1715TC+5.5100$ | 0.69 | 5.138 | 10.8 |
| *C.v.* + *M.c.* | $SPAD=$ 0.84679TC + 10.31619 | 0.69 | 5.155 | 10.8 |

**Supplementary table S6.** Equations for the best fitting and linear models for Laminar leaves shown in **Figure 4**. TC – total chlorophyll in μg cm^-2^; CM = chlorophyll meter; coefficients of determination (R^2^), root mean square error (RMSE) and RMSE normalized by the variable range (nRMSE in %)

| **Chlorophyll meter** | **Model** |  | **R^2^** | **RMSE  [CM values]** | nRMSE% |
| --- | --- | --- | --- | --- | --- |
| CCM 300 | Best fitting | ${CCM}_{CFR}= -0.0002{TC}^{2}+0.0267TC+0.5900$ | 0.92 | 0.092 | 6.6 |
|  | Linear | ${CCM}_{CFR}= 0.0136TC+0.7554$ | 0.84 | 0.133 | 9.6 |
| SPAD | Best fitting | $SPAD= -0.0026{TC}^{2}+1.0587TC+7.4766$ | 0.86 | 7.585 | 8.9 |
|  | Linear | $SPAD= 0.8577TC+10.1521$ | 0.86 | 7.698 | 9.0 |
| Dx | Best fitting | $Dx= -0.0008{TC}^{2}+0.7466TC+4.7739$ | 0.89 | 5.430 | 8.2 |
|  | Linear | $Dx= 0.6823TC+5.5871$ | 0.89 | 5.437 | 8.2 |
| MSPQ | Best fitting | $MSPQ= -0.0077{TC}^{2}+1.4664TC+7.5695$ | 0.86 | 8.444 | 10.5 |
|  | Linear | $MSPQ= 0.8891TC+14.5543$ | 0.82 | 9.475 | 11.7 |

**Supplementary table S7.** Equations for the best fitting and linear models for Laminar leaves shown in **Figure 4**. TC – total chlorophyll in μg cm^-2^. In case of VI Vogelmann, the quadratic coefficient is very low, thus the quadratic term is negligible and RMSE equals to the one of linear model. Coefficients of determination (R^2^), root mean square error (RMSE) and RMSE normalized by the variable range (nRMSE in %).

| **Spectral Index** | **Model** |  | **R^2^** | **RMSE  [index values]** | **nRMSE%** |
| --- | --- | --- | --- | --- | --- |
| Vogelmann | Best fitting | $Vogelm= -0.0000{TC}^{2}+0.0111TC+1.0142$ | 0.95 | 0.057 | 5.4 |
|  | Linear | $Vogelm= 0.0107TC+1.0196$ | 0.95 | 0.057 | 5.4 |
| NDchl | Best fitting | $NDchl= -0.0001{TC}^{2}+0.0111TC+0.0364$ | 0.95 | 0.039 | 6.0 |
|  | Linear | $NDchl= 0.0071TC+0.0873$ | 0.91 | 0.049 | 7.5 |
| Datt2 | Best fitting | $Datt2= 0.0006{TC}^{2}+0.0307TC+1.0275$ | 0.94 | 0.211 | 5.8 |
|  | Linear | $Datt2= 0.0357TC+0.9646$ | 0.93 | 0.213 | 5.9 |
| RMSR | Best fitting | $RMSR= -0.0001{TC}^{2}+0.02924TC+0.0397$ | 0.93 | 0.129 | 6.8 |
|  | Linear | $RMSR= 0.0208TC+0.1461$ | 0.91 | 0.143 | 7.5 |

**Supplementary table S8:** Biophysical traits for laminar leaves according to their three leaf types regarding leaf anatomy: mesomorphic, scleromorphic and scleromorphic with hypodermis (HY). Leaf mass per area (LMA), equivalent water thickness (EWT), chlorophyll, carotenoids, and anthocyanin contents. Chlorophyll and carotenoids are expressed also in nanomolar area-based units, which was necessary to assess anthocyanin to chlorophyll ratio for further analyses. s.d. = standard deviation

|  |  | **LMA**  **mg . cm^-2^** | **EWT**  **(mg . cm^-2^)** | **Chlorophyll µg.cm^-2^** | **Carotenoids µg.cm^-2^** | **Chlorophyll nmol.cm^-2^** | **Carotenoids nmol.cm^-2^** | **Anthocyanins n mol.cm^-2^** |
| --- | --- | --- | --- | --- | --- | --- | --- | --- |
| **Mesomorphic** | mean | 4.59 | 8.80 | 25.17 | 4.17 | 28.04 | 7.32 | 25.51 |
|  | s.d. | 1.71 | 3.17 | 17.09 | 2.05 | 19.03 | 3.60 | 26.50 |
|  | min | 1.96 | 4.08 | 0.24 | 0.72 | 0.27 | 1.26 | 2.35 |
|  | max | 9.52 | 16.81 | 68.21 | 11.20 | 75.94 | 19.65 | 154.12 |
|  | range | 7.56 | 12.72 | 67.97 | 10.48 | 75.67 | 18.39 | 153.65 |
|  | n | 112 | 112 | 112 | 112 | 112 | 112 | 110 |
| **Scleromorphic** | mean | 9.45 | 15.82 | 43.99 | 5.41 | 49.01 | 9.50 | 19.04 |
|  | s.d. | 3.59 | 3.91 | 25.01 | 2.51 | 27.87 | 4.41 | 18.95 |
|  | min | 1.51 | 1.43 | 1.30 | 1.89 | 1.45 | 3.32 | 0.69 |
|  | max | 20.49 | 24.79 | 100.88 | 12.24 | 112.45 | 21.47 | 76.57 |
|  | range | 18.98 | 23.36 | 99.58 | 10.34 | 111.01 | 18.15 | 75.88 |
|  | n | 56 | 56 | 56 | 56 | 56 | 56 | 54 |
| **Scleromorphic - HY** | mean | 6.19 | 15.69 | 53.18 | 6.28 | 59.25 | 11.03 | 11.54 |
|  | s.d. | 2.59 | 3.40 | 15.44 | 2.00 | 17.21 | 3.51 | 9.49 |
|  | min | 3.12 | 11.43 | 15.47 | 1.34 | 17.23 | 2.35 | 1.61 |
|  | max | 12.66 | 23.26 | 74.33 | 8.81 | 82.82 | 15.46 | 38.39 |
|  | range | 9.54 | 11.83 | 58.86 | 7.47 | 65.59 | 13.11 | 36.78 |
|  | n | 25 | 25 | 25 | 25 | 25 | 25 | 24 |

**Supplementary Table S9:** Detailed anatomical traits for laminar leaves according to their three leaf types regarding leaf anatomy: mesomorphic, scleromorphic and scleromorphic with hypodermis (HY). Leaf mass per area (LMA) and thickness of the leaf and individual tissue layers from adaxial to abaxial surface. s.d. = standard deviation.

|  |  | **LMA**  **mg . cm^-2^** | **Leaf Thickness**  **µm** | **Palisade parenchyma µm** | **Spongy parenchyma µm** | **Abaxial epidermis µm** | **Adaxial epidermis µm** |
| --- | --- | --- | --- | --- | --- | --- | --- |
| **Mesomorphic** | mean | 4.59 | 114.40 | 34.10 | 48.72 | 11.55 | 16.08 |
|  | s.d. | 1.71 | 19.46 | 9.74 | 10.31 | 1.85 | 2.77 |
|  | min | 1.96 | 83.13 | 21.41 | 31.83 | 8.02 | 10.44 |
|  | max | 9.52 | 166.95 | 64.95 | 82.26 | 15.42 | 22.98 |
|  | range | 7.56 | 83.81 | 43.54 | 50.43 | 7.40 | 12.54 |
|  | n | 112 | 38 | 38 | 38 | 38 | 38 |
| **Scleromorphic** | mean | 9.45 | 375.1 | 107.03 | 210.97 | 16.89 | 33.79 |
|  | s.d. | 3.59 | 151.25 | 60.34 | 69.50 | 6.85 | 16.93 |
|  | min | 1.51 | 202.34 | 24.63 | 122.32 | 12.24 | 17.26 |
|  | max | 20.49 | 648.39 | 36.31 | 335.64 | 30.58 | 64.38 |
|  | range | 18.98 | 446.05 | 208.86 | 213.31 | 18.34 | 47.12 |
|  | n | 56 | 6 | 6 | 6 | 6 | 6 |
| **Scleromorphic - HY** | mean | 6.19 | 288.39 | 34.72 | 174.34 | 23.28 | 49.64 |
|  | s.d. | 2.59 | 107.19 | 7.97 | 99.98 | 4.75 | 4.65 |
|  | min | 3.12 | 26.80 | 21.14 | 71.62 | 17.66 | 42.35 |
|  | max | 12.66 | 170.78 | 54.22 | 319.47 | 32.64 | 57.97 |
|  | range | 9.54 | 275.03 | 33.08 | 247.85 | 14.98 | 15.63 |
|  | n | 25 | 16 | 16 | 16 | 16 | 16 |

**Supplementary table S10.** Linear equations, which inversion was used for total chlorophyll content calculation of validation samples in Figure 10. TC – total chlorophyll in μg cm^-2^; CM = chlorophyll meter; coefficients of determination (R^2^); root mean square error (RMSE) and RMSE normalized by the variable range (nRMSE in %).

| **Chlorophyll meter** | **Linear model (only laminar mesomorphic leaves)** | **R^2^** | **RMSE [CM values]** | **nRMSE%** |
| --- | --- | --- | --- | --- |
| CCM 300 | ${CCM}_{CFR}= 0.0166TC+0.6693$ | 0.87 | 0.112 | 9.5 |
| SPAD | $SPAD= 0.7677TC+9.2786$ | 0.86 | 5.118 | 8.5 |
| Dx | $Dx= 0.6098TC+5.4378$ | 0.93 | 2.905 | 6.5 |
| MSPQ | $MSPQ= 0.820TC+10.2082$ | 0.87 | 6.594 | 9.2 |

**Supplementary table S11.** Linear equations, which inversion was used for total chlorophyll content calculation of validation samples in Figure 11. TC – total chlorophyll in μg cm^-2^; coefficients of determination (R^2^); root mean square error (RMSE) and RMSE normalized by the variable range (nRMSE in %).

| **Vegetation Index** | **Linear model (only laminar mesomorphic leaves)** | **R^2^** | **RMSE [index values]** | **nRMSE%** |
| --- | --- | --- | --- | --- |
| Vogelmann | $Vogelm= 0.0100TC+1.0245$ | 0.94 | 0.044 | 6.8 |
| NDchl | $NDchl= 0.0079TC+0.0619$ | 0.92 | 0.040 | 8.0 |
| Datt2 | $Datt2= 0.0308TC+1.0328$ | 0.93 | 0.140 | 7.0 |
| RMSR | $RMSR= 0.0215TC+0.1024$ | 0.92 | 0.108 | 8.0 |

**Supplementary table S12.** Root mean square error (RMSE in µg.cm^-2^) for all tested chlorophyll meters, VIs and three types of models. NA = not available in case that the polynomial inversion caused many invalid predictions and RMSE was not calculated. In case of polynomial models few predictions were not valid (up to six) and thus those were omitted before calculating RMSE.

|  | Chlorophyll meter | | | | Vegetation indices | | | |
| --- | --- | --- | --- | --- | --- | --- | --- | --- |
| Model type | **CCM300** | **SPAD** | **Dx** | **MSPQ** | **Vogelmann** | **NDchl** | **Datt2** | **RMSR** |
| Linear | 9.76 | 8.85 | 10.52 | 6.59 | 6.77 | 7.02 | 7.10 | 7.56 |
| Logarithmic | 65.16 | 26.47 | 657.39 | 5.58 | 165.72 | 64.31 | 451.05 | 87.72 |
| Polynomial | NA | 8.64 | 12.38 | 7.33 | 7.11 | *7.12* | 7.03 | *8.42* |

**Supplementary Table S13:** Descriptive statistics for training and validation dataset on laminar leaves for laboratory (spectrophotometrically) determined Chl content; Optically-determined chlorophyll content - Chl_opt_; laboratory-determined ‘absolute’ chlorophyll content - Chl_abs_. Values of chlorophyll content determined by different chlorophyll meters: SPAD_values_, Dx_values_, MSPQ_values_, CCM_values_ and four chlorophyll vegetation indices: Vogelmann, NDchl, Datt2 and RMSR – for their detailed description see the Supplementary Table S19.

| ***Laminar leaves*** | ***Dataset*** | ***Chlorophyll (µg.cm^-2^)*** | ***CCM_CFR_*** | ***SPAD_values_*** | ***Dx_values_*** | ***MSPQ_values_*** | ***Vogelmann*** | ***NDchl*** | ***Datt2*** | ***RMSR*** |
| --- | --- | --- | --- | --- | --- | --- | --- | --- | --- | --- |
| Mean | Training | 34.26 | 1.22 | 40.15 | 28.96 | 48.18 | 1.38 | 0.33 | 2.19 | 0.86 |
|  | Validation | 34.44 | 1.23 | 32.99 | 31.37 | 38.13 | 1.35 | 0.33 | 2.07 | 0.78 |
| Standard deviation | Training | 22.36 | 0.33 | 20.40 | 16.19 | 21.46 | 0.25 | 0.17 | 0.83 | 0.38 |
|  | Validation | 18.41 | 0.31 | 12.19 | 12.90 | 15.18 | 0.18 | 0.13 | 0.60 | 0.38 |
| min | Training | 0.24 | 0.41 | 0.50 | 1.47 | 1.18 | 0.74 | 0.00 | 1.00 | -0.13 |
|  | Validation | 0.36 | 0.43 | 0.40 | 2.40 | -2.15 | 1.01 | 0.02 | 1.03 | 0.02 |
| max | Training | 100.88 | 1.80 | 86.20 | 67.80 | 78.78 | 2.06 | 0.65 | 4.61 | 1.91 |
|  | Validation | 79.58 | 1.85 | 58.37 | 63.47 | 66.11 | 1.81 | 0.59 | 3.83 | 1.66 |
| range | Training | 100.65 | 1.39 | 85.70 | 66.33 | 77.60 | 1.33 | 0.65 | 3.61 | 2.04 |
|  | Validation | 79.22 | 1.42 | 57.97 | 61.07 | 68.26 | 0.80 | 0.57 | 2.80 | 1.65 |
| n | Training | 193 | 192 | 189 | 193 | 133 | 193 | 193 | 193 | 193 |
|  | Validation | 233 | 232 | 232 | 203 | 179 | 233 | 233 | 233 | 233 |

**Supplementary Table S14:** Locations in Czech Republic and sampling dates for leaves used for Chl (biochemical and optical) and anatomical analyses.

| Leaf anatomical type | Location | Altitude  (m a.s.l.) | DOY |
| --- | --- | --- | --- |
| Laminar leaves: mesomorphic, scleromorphic,  scleromorphic - HY | Botanical garden of Charles University, Prague, central Czechia | 220 | 2019: 119, 203, 259, 294 |
| Laminar leaves:  mesomorphic | Floodplain forest Lanžhot, south-east Czechia | 150 | 2019: 115, 199, 255, 290  2020: 132, 209, 296 |
| Grass leaves | Relict alpine-arctic grassy tundra, Krkonoše Mts., north Czechia | 1410 | 2020: 168, 196, 223 |
| Needles | Bílý Kříž experimental station, Beskydy Mts., north-east Czechia | 890 | 2020: 205, 266  2021: 145, 229, 285 |

**Supplementary Figure S15**

**
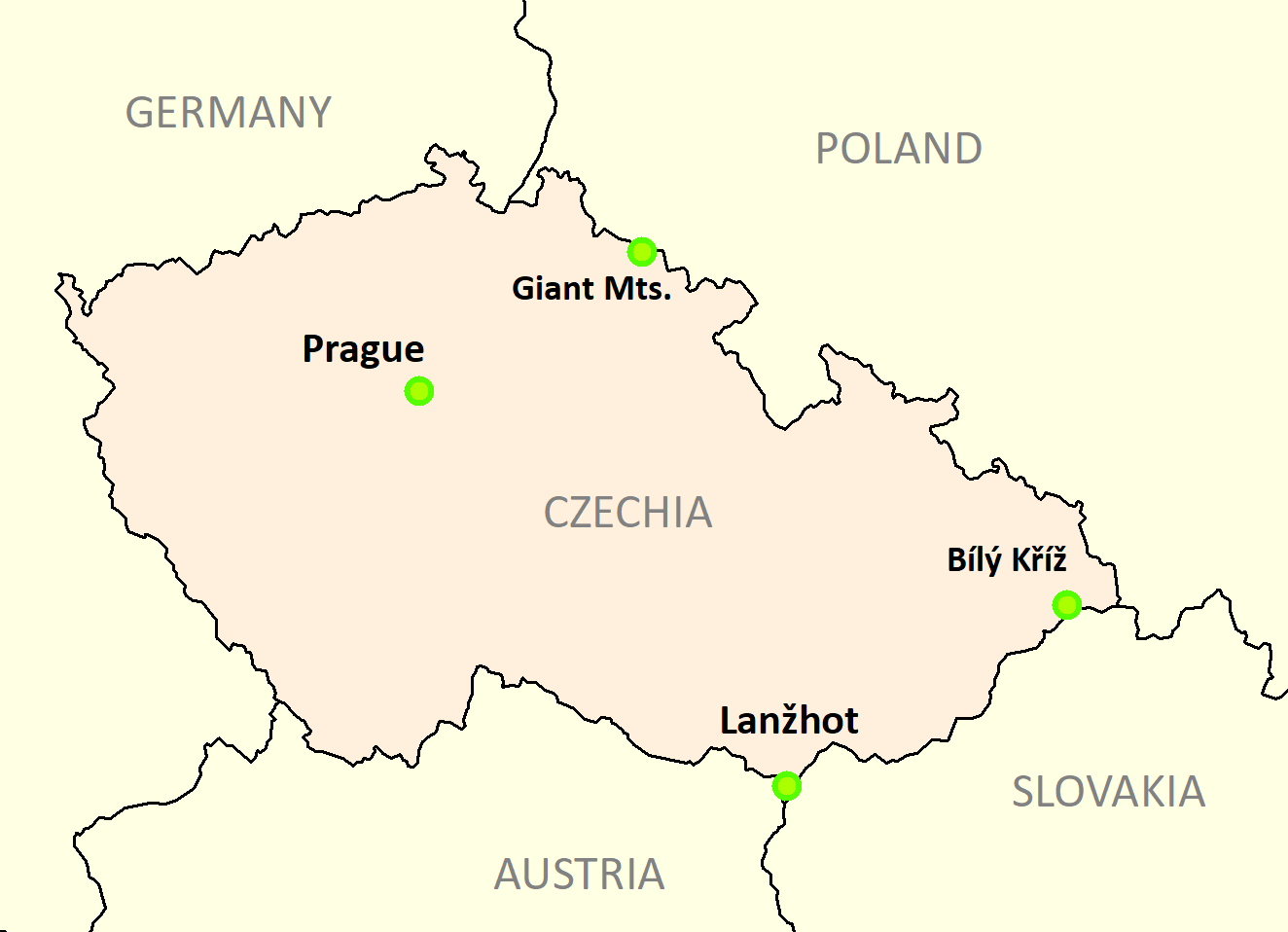
**

**Supplementary Figure S17:** The map of sample collection locations: Laminar leaves (Prague – Botanical garden, Lanžhot – mixed floodplain forest), Grass leaves (Giant Mts. – relict arctic-alpine grassland) and Needles (Norway spruce forest).

**Supplementary Table S16:** Woody plants with laminar dorsiventral leaves used in the study. Database number of sampled individuals from the Botanical Garden, Charles University Science Faculty, Prague. The formal identification of species for the database undertook dendrologists and botanists T. Vencálek, Z. Šípek and T. Procházka. The individuals without the database number were sampled in the Faculty of Science campus and the identification was confirmed by Mgr. T. Procházka.

| Laminar leaves - Species | Family | Database number |
| --- | --- | --- |
| Subgroup mesomorphic | |  |
| *Acer campestre* L. | *Sapindaceae* | PE000083 |
| *Acer palmatum* Thunb. | *Sapindaceae* | PE000044 |
| *Acer platanoides* L. | *Sapindaceae* | *-* |
| *Carpinus betulus* 'Pendula' | *Betulaceae* | PE000007 |
| *Corylus maxima* 'Rubra' | *Betulaceae* | PE000817 |
| *Magnolia* sp. L. | *Magnoliaceae* | *-* |
| *Malus x purpurea* (Barbier) Rehd. | *Rosaceae* | PE000243 |
| *Parthenocissus tricuspidate* ([Siebold](https://cs.wikipedia.org/w/index.php?title=Sieb.&action=edit&redlink=1) et [Zucc.](https://cs.wikipedia.org/w/index.php?title=Zucc.&action=edit&redlink=1)) [Planch.](https://cs.wikipedia.org/w/index.php?title=Planch.&action=edit&redlink=1) | *Vitaceae* | *-* |
| *Quercus robur* 'Fastigiata' | *Fagaceae* | PE000317 |
| *Syringa vulgaris* L. | *Oleaceae* | PE000356 |
| *Tilia × euchlora* K. Koch | *Malvaceae* | PE000210 |
| Subgroup scleromorphic | |  |
| *Hedera helix* L. | *Araliaceae* | *-* |
| *Mahonia aquifolium* ([Pursh](https://cs.wikipedia.org/w/index.php?title=Frederick_Traugott_Pursh&action=edit&redlink=1)) [Nutt.](https://cs.wikipedia.org/w/index.php?title=Thomas_Nuttall&action=edit&redlink=1) | *Berberidaceae* | *-* |
| *Prunus laurocerasus* L. | *Rosaceae* | PE000092 |
| *Rhododendron* × praecox | *Ericaceae* | PE000520 |
| Subgroup scleromorphic - HY | |  |
| *Ficus benjamina* L. | *Moraceae* | TR001019 |
| *Ficus lyrata* Warb*.* | *Moraceae* | TR001622 |
| *Ficus natalensis subsp. leprieurii* (Miq.) C.C.Berg | *Moraceae* | TR000665 |

**Supplementary Table S17:** Vegetation indices used in the model training and independent validation to Chl_abs_ and Chl_opt_, where R indicates reflectance in given wavelength. Optically determined chlorophyll content - Chl_opt_; laboratory-determined ‘absolute’ chlorophyll content - Chl_abs_.

| Abbreviation | Name of index | Formula | Related to | Scale | Citation |
| --- | --- | --- | --- | --- | --- |
| Vogelmann | [Simple Ratio 740/720 hyper Vogelmann indices 1](https://www.indexdatabase.de/db/i-single.php?id=100) | R_740_/X_720_ | Chlorophyll | Leaf | ^1^ |
| Datt2 | [Simple Ratio 850/710 Datt2](https://www.indexdatabase.de/db/i-single.php?id=151) | R_850_/R_710_ | Chlorophyll | Leaf | ^2^ |
| NDchl | Normalized Difference Chlorophyll | (R_925_-R_710_)/(R_925_+R_710_) | Chlorophyll | Canopy | ^3^ |
| RMSR | Revised Modified Simple Ratio | ((R_750_/R_705_) -1)/sqrt((R_750_/R_705_)+1) | Chlorophyll | Model simulation | ^4^ |

**References**

1. Vogelmann, J. E., Rock, B. N. & Moss, D. M. Red edge spectral measurements from sugar maple leaves. *International Journal of Remote Sensing* **14**, 1563–1575 (1993).

2. Datt, B. A New Reflectance Index for Remote Sensing of Chlorophyll Content in Higher Plants: Tests using Eucalyptus Leaves. *Journal of Plant Physiology* **154**, 30–36 (1999).

3. Lemaire, G. *et al.* Calibration and validation of hyperspectral indices for the estimation of broadleaved forest leaf chlorophyll content, leaf mass per area, leaf area index and leaf canopy biomass. *Remote Sensing of Environment* **112**, 3846–3864 (2008).

4. Wu, C., Niu, Z., Tang, Q. & Huang, W. Estimating chlorophyll content from hyperspectral vegetation indices: Modeling and validation. *Agricultural and Forest Meteorology* **148**, 1230–1241 (2008).
